# A bottleneck-first evaluation of antigen delivery platforms for therapeutic cancer vaccines

**DOI:** 10.1016/j.mtbio.2026.103715

**Published:** 2026-09-30

**Authors:** Shuai Wang, Mai Zhang, Jing Tian, Jiong Wu, Shentao Lu, Lubin Liu, Dingkang Liu

**Affiliations:** a Department of Obstetrics and Gynecology, Women and Children's Hospital of Chongqing Medical University, Chongqing, 400013, China; b Department of Obstetrics and Gynecology, West China Hospital, Sichuan University, Chengdu, Sichuan, 610041, China; c Jiangsu Key Laboratory of Druggability of Biopharmaceuticals and State Key Laboratory of Natural Medicines, School of Life Science and Technology, China Pharmaceutical University, Nanjing, 210009, China

**Keywords:** Therapeutic cancer vaccine, Antigen delivery, Biomaterial carrier, Delivery bottleneck, Structure–function relationship, Cancer immunotherapy

## Abstract

Therapeutic cancer vaccines require tumor-relevant antigens and delivery systems that preserve antigen integrity, control tissue and cellular exposure, support productive processing, and sustain antigen-specific immunity. Yet platform performance cannot be inferred from material properties, cellular uptake, or tumor control alone, because these endpoints do not identify which delivery step changed or whether that change contributed to an antigen-specific response. Seven material matrices and a separate carrier-free molecular assembly mode are evaluated across four functional domains. A carrier advantage is supported only when a defined variable changes a prespecified delivery function under matched conditions, a matched delivery endpoint captures that change, and the result is linked to an antigen-specific outcome; selected variable-to-outcome chains are appraised qualitatively using four possible categories: supported, partial, untested, or refuted. Repeat-dose behavior, carrier-directed immunity, routine lot release, manufacturing consistency, and comparability following manufacturing process changes are treated as additional constraints because they can modify or confound an initial advantage. Artificial intelligence-assisted design is considered only for hypothesis triage and validation planning. The reviewed evidence does not support a universal platform hierarchy. Platform selection should instead begin with the dominant biological or product-level bottleneck.

## Highlights

• Current evidence does not support a universal carrier hierarchy across bottlenecks.
• A carrier advantage needs matched delivery evidence and an antigen-specific response.
• Formulation benefits can coexist with unresolved carrier-specific attribution.
• Carrier immunity matters only if delivery, vaccine response, or tolerability changes.
• Artificial intelligence ranks candidates; four experimental gates govern advancement.

## Graphical abstract

**Figure:**
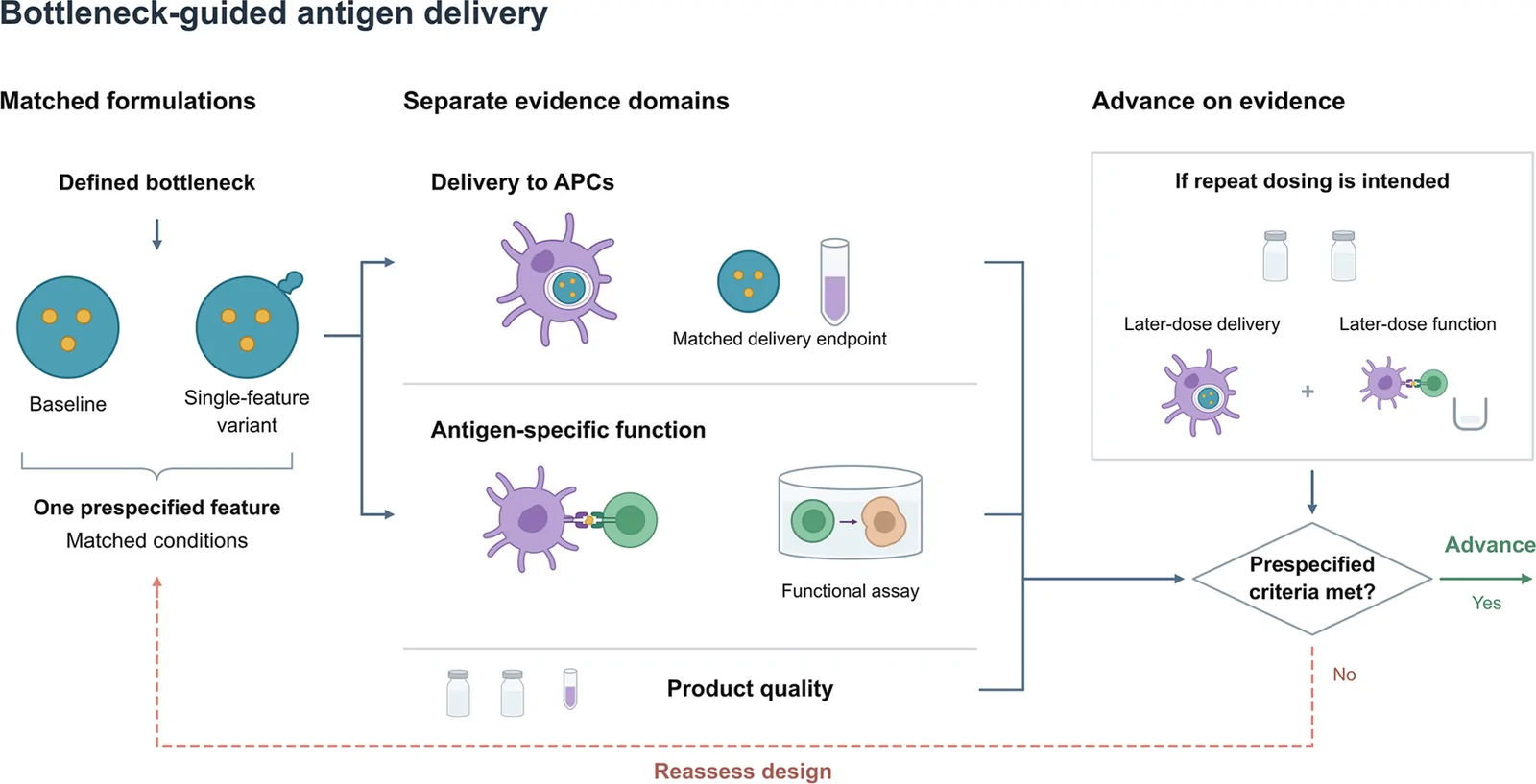

## 1 Introduction: delivery design as a bottleneck in therapeutic cancer vaccination

Therapeutic cancer vaccines now span shared tumor antigens and patient-specific neoantigens. Personalized regimens show that selected antigens can prime tumor-reactive T cells [1,2], and recent clinical studies support their expansion and persistence [[3], [4], [5]]. A vaccine must still protect its antigen, reach the relevant antigen-presenting cells (APCs), enter productive processing pathways, and retain function across later doses without unfavorable changes in pharmacokinetics, biodistribution, or immunodominance.

Antigen quality includes tumor specificity, clonality, expression, processing, and human leukocyte antigen (HLA) presentation potential [[6], [7], [8]]. Even a strong candidate can fail when it is unstable, fails to reach lymphoid tissues, enters an unsuitable APC subset, or is degraded without adequate co-stimulation [[9], [10], [11]]. Primed T cells then face stromal extracellular matrix, abnormal vasculature, regulatory T cells, suppressive myeloid populations, and metabolically suppressive conditions in tumors [[12], [13], [14]]. Increased carrier uptake is therefore not sufficient evidence of improved vaccination.

Existing reviews examine cancer-vaccine nanotechnology [15,16], protein antigen-display platforms [17], delivery technologies across vaccine formats [18], and nano–bio interface engineering for protein delivery [19]. Broader reviews address vaccine development, immunological evaluation, and formulation [[20], [21], [22], [23], [24]]. Wusiman et al. examine biomimetic nanovaccines through their immune mechanisms, platform design, preclinical efficacy, and clinical translation [25]. Li et al. relate the composition and structure of nanoengineered coordination polymers to immune-related payload protection and cancer immunotherapy [26]. Liang et al. organize biomaterial strategies around activation of the cyclic GMP–AMP synthase–stimulator of interferon genes pathway [27]. These reviews provide complementary accounts of material design and immune mechanisms.

The present review complements these perspectives through a qualitative appraisal of specific carrier claims across material classes. Bottleneck-first evaluation begins with a defined biological or product-level limitation, then asks whether a controllable carrier variable changes a matched delivery endpoint and an antigen-specific functional outcome. Selected comparisons are assessed using four possible categories: supported, partial, untested, or refuted (Section 5.4 and Table 5). The analysis separates demonstrated formulation benefits from unresolved attribution to individual carrier features and identifies limits to transferring those benefits to other designs. Evidence for a clinical vaccine regimen and constraints imposed by repeat dosing and product quality provide further context (Sections 2.4, 5.3). Modular bacteriophage and bacterial hydrogel systems illustrate controllable behavior [28,29], but their platform labels alone do not establish a delivery benefit.

A core platform is defined as an administered, non-replicating material or biologically derived carrier engineered to change antigen protection, transport, entry, release, processing, or presentation. Immune modulation is included only when material-defined spatial or temporal coupling to vaccination is verified and linked to antigen-specific function. Systemic checkpoint blockade, antigen-independent local immunotherapy, replication-competent oncolytic viruses, and ex vivo cellular products are boundary comparators. *In situ* vaccination is included only when a material controls antigen availability, APC access, or presentation.

*Programmable* denotes a modular change that produces a prespecified, reproducible delivery change with relevant confounders controlled; tunability or computational ranking alone is insufficient. Immune modulation qualifies only when its coupling to antigen and antigen dependence are demonstrated. The four functional domains are pre-uptake interfaces, payload relationships, release and intracellular transfer, and antigen organization. *Carrier visibility* denotes recognition of carrier components distinct from the intended antigen and remains separate from carrier-intrinsic innate activity. Repeat dosing, routine lot release, manufacturing consistency, and post-change comparability are evaluated alongside delivery. Matrix classification serves as descriptive vocabulary rather than a platform-selection rule.

## 2 Material classes, evidence maturity, and translational constraints

### 2.1 Classification by material matrix and assembly mode

Classification uses two axes. The material axis comprises lipid, polymer, coordination network, other inorganic or mineral matrix, biological membrane or vesicle, protein scaffold or capsid, and nucleic acid nanostructure. The assembly/interface axis includes carrier-free molecular assembly, in which antigen and, when applicable, adjuvant assemble without a separate carrier matrix, as well as ligands, responsive bonds, and coatings. Hybrids should report both core and interface modules; metal–organic frameworks (MOFs) are coordination networks. The shared variables, functions, and constraints are compared across the seven matrices and the carrier-free molecular assembly mode in Table 1.

**Table 1:** Material matrices and the carrier-free molecular assembly mode.

Matrix or assembly mode	Representative forms	Main engineerable variables	Directly supported delivery functions	Main interpretive constraint
Lipid [[30], [31], [32]]	LNP, liposome	Lipid identity and structure, component ratio, apparent pK_a_ of the formulated particle (when ionizable lipids are present), PEG–lipid, ligand, mRNA format	Nucleic acid protection, transfection, tissue exposure, association with immune cells	Innate immune activation, anti-PEG antibody responses, formulation-dependent tropism
Polymer [33,34]	Degradable nanoparticle, dendrimer, polymersome	Polymer chemistry, charge, hydrophobicity, degradation, linker	Sustained release, co-localization, intracellular transfer	Chemistry-dependent loading, release, and membrane interaction
Coordination network [35,36]	MOFs and other metal–ligand coordination networks	Metal–ligand chemistry, pore architecture, responsive bond, surface ligand	Cargo loading, stimulus-coupled release, local immune modulation	Metal-dependent toxicity, degradation, product heterogeneity
Other inorganic or mineral matrix [33,37,38]	Mineralized or metal oxide particle	Composition, ion release, surface chemistry, porosity	Adjuvant delivery, responsive release, imaging-coupled localization	Persistence, ion toxicity, uncertain biodegradation
Biological membrane or vesicle [39,40]	EV, cell-derived vesicle; membrane-coated hybrids classified by core matrix	Source cell, membrane identity, cargo loading, isolation, storage	Source-dependent trafficking, cell interaction, biomimetic display	Source heterogeneity, contaminants, difficult mechanistic attribution
Protein scaffold or capsid [17,41,42]	Protein cage, VLP, de novo scaffold	Valency, spacing, orientation, linker, assembly state	Ordered antigen display, uptake by APCs, modular reuse	Scaffold immunity, possible MHC presentation and immunogenicity of junction-derived peptides, assembly reproducibility
Nucleic acid nanostructure [[43], [44], [45]]	DNA origami	Geometry, strand composition, attachment site, nuclease protection	Defined antigen organization and spatial control	Innate DNA sensing, stability, cost, scale
Carrier-free molecular assembly [46]	Peptide–adjuvant conjugate, amphiphilic antigen assembly	Stoichiometry, amphiphilicity, sequence, adjuvant integration	Antigen–adjuvant coupling with reduced inert-carrier burden	Sequence-dependent assembly, release, and product consistency

*Note:* References identify representative designs and delivery evidence; the listed constraints are this review's synthesis, not properties shared by every formulation. Protein-geometry and DNA-sensing sources include non-cancer contexts.

*Abbreviations:* APC, antigen-presenting cell; EV, extracellular vesicle; LNP, lipid nanoparticle; MHC, major histocompatibility complex; mRNA, messenger RNA; MOF, metal–organic framework; PEG, polyethylene glycol; VLP, virus-like particle.

Lipid nanoparticles (LNPs) and liposomes vary in lipid composition, component ratios, polyethylene glycol–conjugated lipid (PEG–lipid), ligands, and messenger RNA (mRNA) format [30,[47], [48], [49], [50]]; cancer-vaccine studies illustrate the formulation dependence of these variables [[51], [52], [53], [54]]. Ionizable formulations add lipid identity, structure, and formulation-dependent apparent pK_a_ [55,56]. In a direct formulation comparison, 113-O12B shifted expression toward draining lymph nodes and away from the liver, but the comparison did not isolate lipid identity from other formulation features [31]. Composition-dependent innate immune activation likewise requires formulation-specific interpretation [32,57]. Selective organ targeting was demonstrated for the liver, lung, and spleen, not for lymph-node targeting or therapeutic cancer vaccination [58]. V940 (mRNA-4157) provides randomized human evidence for an LNP-formulated vaccine regimen, but the trial evaluates addition of the whole vaccine rather than an isolated carrier variable [2].

Polymer matrices vary in chemistry, charge, degradation, and linker design [33,59,60]. Therapeutic labels do not determine matrix class. Calcium phosphate is an inorganic or mineral matrix [37], the membrane-derived vaccine belongs to the biological membrane or vesicle group [61], and the phenylboronic ester–modified formulation is polymeric [34]. MOFs belong to coordination networks [35], whereas MnO_2_ is an inorganic matrix [38]. Additional examples include a carrier-free system [62], a manganese-coordinated network [36], and a biomineralized system [63]. Photoresponsive, ultrasound-responsive, or immune checkpoint–modulating systems do not by themselves establish delivery of a selected exogenous antigen [[64], [65], [66], [67]].

Membranes and extracellular vesicles (EVs) inherit source-cell interfaces and batch constraints [39,40,[68], [69], [70]]. Protein-based antigen-presentation platforms support multivalent display [17]. De novo studies establish defined cage architectures [41,[71], [72], [73]], while separate tumor and viral vaccine studies illustrate protein-cage or VLP applications [42,[74], [75], [76]]; scaffold and repeat-dose constraints are discussed in Section 5.1. Nucleic acid structures offer spatial control with distinct sensing and scale constraints [[43], [44], [45]]. Peptide–adjuvant conjugates can assemble without a separate carrier [46]. A protein-delivering dendrimer is polymeric [77], whereas a co-localized nanomedicine–vaccine regimen operates at the tissue level rather than through carrier-level linkage [78].

Different matrices can reach the same function through different variables and risks. Matrix selection follows the bottleneck-first framework introduced above (Fig. 1). Evidence maturity and attribution should therefore be considered alongside repeat-dose and manufacturing constraints, without implying a universal platform ranking (Table 2A, Table 2BA and 2B).

**Fig. 1:**
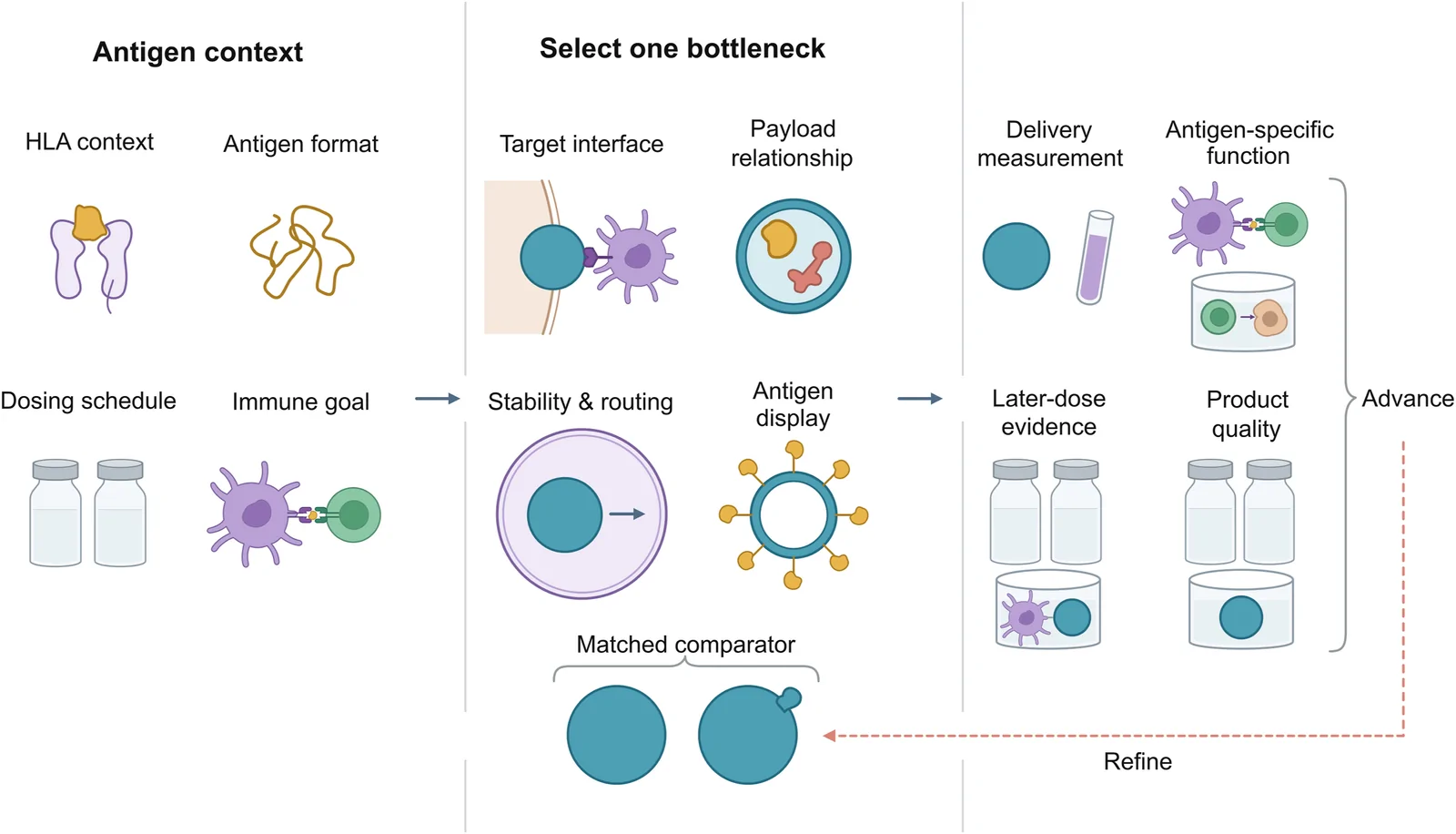
Matched comparisons and evidence gates for antigen-delivery candidates. From left to right, antigen context comprises the human leukocyte antigen (HLA) context, antigen format, dosing schedule, and immune goal. One of four non-ranked bottlenecks—target interface, payload relationship, stability and routing, or antigen display—is then tested against a matched comparator. Advancement requires delivery measurement, antigen-specific function, later-dose evidence when repeat dosing is intended, and product quality; a measured shortfall returns the candidate to refinement. Created in BioRender. Wang, S. (2026) https://BioRender.com/1meeco7.

### 2.2 Evidence maturity and attribution boundaries

**Table 2A:** Evidence maturity and attribution boundaries across platforms.

Platform, material matrix, or assembly mode	Cited evidence and product context	Attribution boundary
Lipid nanoparticle (LNP)	Human evidence on anti-PEG antibodies is available [79], and a separate study provides pharmacokinetic evidence after a single booster dose [80]. Other studies describe small-scale neoantigen-encoding mRNA and parallelized LNP production processes [81,82]. INTerpath-001 is a phase III trial of V940 plus pembrolizumab (NCT05933577) [83]; sponsors announced that its recurrence-free-survival and distant-metastasis-free-survival endpoints were met on 19 August 2026, without disclosing phase III effect estimates [84]. In KEYNOTE-942 (n = 157), the primary recurrence-free survival analysis was positive under the prespecified one-sided α = 0.10 criterion (hazard ratio, 0.561; 95% confidence interval, 0.309–1.017; two-sided P = 0.053); the 5-year analyses of recurrence-free survival and distant metastasis-free survival were descriptive, and overall survival data remained immature [2,85].	V940 and pembrolizumab were administered separately, so the regimen neither tests physical co-delivery nor isolates an LNP variable. Process studies do not establish V940 manufacturing performance or access.
Polymer	Cancer-vaccine evidence from broad polymer or nanoparticle studies is predominantly preclinical [33,59,60]. Antigen-delivery examples are also preclinical [34,77]. The polymersome study delivered a stimulator of interferon genes (STING) agonist, not a selected antigen [86].	Chemistry, cargo, and endpoints differ; agonist routing or tumor regression cannot establish selected-antigen delivery.
Coordination network	Cited MOF and manganese-coordinated studies are preclinical [35,36]. Photoresponsive and locally immunomodulatory systems are also preclinical boundary comparators [[64], [65], [66], [67]].	Triggered release or local immune activation alone does not establish antigen-specific delivery.
Other inorganic or mineral matrix	The cited calcium phosphate, manganese dioxide, and biomineralized systems are preclinical [37,38,63].	Composition-specific ion release and innate immune activity preclude a class-wide inference from tumor control or APC activation.
Biological membrane or extracellular vesicle (EV)	Source-related evidence spans membrane-derived systems [61] and EV studies [39,40,[68], [69], [70]]. EV identity and product-quality considerations require separate characterization [87,88]. In a 22-patient single-arm dendritic cell–derived exosome maintenance study after platinum chemotherapy and metronomic cyclophosphamide, 4-month progression-free survival was 32% (95% confidence interval, 16%–53%), below the 50% target; median progression-free survival was 2.2 months, with no objective response according to RECIST [89].	The lack of a concurrent control, post-chemotherapy selection, and cyclophosphamide prevent attribution to the exosome alone and do not validate EV carriers as a class.
Protein cage or virus-like particle (VLP)	Cancer-vaccine evidence is largely preclinical. The cited human evidence documents scaffold-specific CD4^+^ T-cell responses in prophylactic HIV vaccination [90]. Pre-existing carrier immunity and scaffold-directed antibody responses have been studied [91,92], as have epitopic suppression and scaffold-directed T-cell responses [[93], [94], [95]]. VLP production studies address product characterization [96,97], while ferritin cancer-vaccine studies remain preclinical [98,99]. A metalloprotein *in situ* vaccine is retained only as a boundary comparator [100], and a non-ferritin protein nanoparticle provides a separate tumor-neoantigen display example [101].	Antibody or CD4^+^ T-cell evidence from prophylactic vaccination does not demonstrate therapeutic cancer efficacy, isolate geometry, or establish preserved booster performance.
DNA nanostructure, including DNA origami	ClinicalTrials.gov exact-term searches found no human DNA-origami vaccine trial by 8 August 2026; cited vaccine evidence is preclinical [[43], [44], [45],^102^].	Plasmid DNA vaccines are a different product class, and precise nanoscale geometry does not establish clinical maturity.
Carrier-free molecular assembly	The cited peptide–adjuvant and amphiphilic antigen assemblies are preclinical [46,62].	Absence of a separate carrier does not establish retained assembly or delivery function after antigen substitution.
Sipuleucel-T, boundary comparator	U.S. Food and Drug Administration (FDA)-approved autologous cellular immunotherapy manufactured ex vivo, with a phase 3 overall survival benefit [103,104].	The cellular manufacturing precedent cannot validate a non-replicating material-delivery platform.
Talimogene laherparepvec (T-VEC), boundary comparator	FDA-approved genetically modified oncolytic viral therapy with phase 3 durable response rate evidence [105,106].	It generates antigen *in situ* rather than carrying a selected exogenous antigen and remains a clinical boundary comparator.

*Note:* Evidence maturity reflects only the cited sources and is not a complete trial inventory.

### 2.3 Repeat-dose, manufacturing, and accessibility constraints

**Table 2B:** Repeat-dose, manufacturing, and accessibility constraints across platforms.

Platform, material matrix, or assembly mode	Persistence and carrier visibility	Later-dose question	Manufacturing, turnaround, or accessibility constraint
Lipid nanoparticle (LNP)	Exposure depends on composition, route, and dose. Formulation-dependent innate activity can vary with ionizable-lipid identity, structure, and proportion and with the broader LNP composition, whereas PEG–lipid components can elicit anti-PEG antibody responses [32,57,79,80].	Measure later-dose exposure, antigen expression, antigen-specific functional response, and tolerability; recognition alone does not establish failure.	Product-level constraints include sequence-specific manufacture, lipid supply, mixing, particle-size distribution or polydispersity, encapsulation or loading, payload integrity, lipid composition, stability, potency, release testing, and treatment-window scheduling [30,81,82]. In KEYNOTE-942, design succeeded for 156/185 tissue-assessed patients (84.3%); among 105 combination-arm patients with successful design, manufacture succeeded for 104 (>99%) [85]. These conditional stage-specific rates do not estimate end-to-end access.
Polymer [33,34]	Chemistry-dependent degradation, retention, charge, hydrophobicity, and membrane activity.	Chemistry-specific later-dose distribution and toxicity data.	Molecular-weight distribution, residuals, loading, release, and potency are product specific.
Coordination network [35,36]	Metal–ligand degradation, ion release, and intrinsic activity may continue after cargo release.	Persistence and repeated exposure require formulation-specific evaluation.	Metal–ligand identity, pore structure, loading, residuals, degradation, release, and potency require joint control.
Other inorganic or mineral matrix [33,37,38]	Ion release and tissue persistence are composition specific.	Accumulation, biodegradation, and later-dose tolerability require product-specific measurement.	Composition, particle-size distribution, morphology, surface state, ion release, and potency require lot control.
Biological membrane or extracellular vesicle (EV)	Source cell, isolation, surface composition, endogenous cargo, and co-isolated non-vesicular material determine fate and immune recognition [39,87,88].	Repeat-dose behavior and responses to source-cell components require product-specific evaluation.	Producer-cell control, isolation, purity, identity, storage, potency, and between-source consistency [40,69,70,87,88].
Protein cage or virus-like particle (VLP)	Non-replicating scaffold; scaffold-specific T-cell and antibody responses have been measured [90,92,95], while pre-existing-carrier effects and epitopic suppression are context dependent [91,93,94].	Test effects on uptake, presentation, immunodominance, potency, and tolerability.	Expression, purification, assembly, antigen copy number, orientation, aggregation, and potency require reproducible lot control [96,97].
DNA nanostructure, including DNA origami	Nuclease degradation, tissue residence, and innate DNA sensing are design dependent [[43], [44], [45]].	Repeat-dose sensing and delivery require design-specific evaluation.	Oligonucleotide identity, stoichiometric assembly, nuclease protection, purification, endotoxin, structure, and scale require control [45].
Carrier-free molecular assembly [46]	Assembly and disassembly are sequence and conjugate dependent; antigen and innate signal may be inseparable.	Antigen replacement can change assembly, exposure, release, and later-dose behavior.	Sequence-specific synthesis, conjugation, stoichiometry, assembly, potency, and product consistency must be re-established.
Sipuleucel-T, boundary comparator [103,104]	Administered cells are not a material carrier.	Three patient-specific doses establish feasibility of repeated treatment.	Leukapheresis, autologous processing, chain of identity, and eligible-site capacity are patient specific.
Talimogene laherparepvec (T-VEC), boundary comparator [105,106]	Replication, intratumoral spread, shedding, clearance, and antiviral immunity differ from non-replicating systems.	Intratumoral redosing cannot be interpreted using LNP, VLP, or protein-cage criteria.	Oncolytic virus manufacturing, infectivity, genetic stability, storage, and intratumoral administration are platform specific.

*Note: Carrier visibility* denotes recognition of non-antigen carrier components. Accessibility also depends on sample adequacy, successful design and release, time to dose, transport and scheduling, eligible-site capacity, and affordability. References support platform properties or product examples. Later-dose questions and proposed product controls are this review's synthesis; the cited studies do not establish repeat-dose performance for every formulation.

*Abbreviations:* APC, antigen-presenting cell; EV, extracellular vesicle; FDA, U.S. Food and Drug Administration; HIV, human immunodeficiency virus; LNP, lipid nanoparticle; mRNA, messenger RNA; MOF, metal–organic framework; PEG, polyethylene glycol; RECIST, Response Evaluation Criteria in Solid Tumors; STING, stimulator of interferon genes; T-VEC, talimogene laherparepvec; VLP, virus-like particle.

### 2.4 Clinical-stage delivery systems and the bottlenecks they address

Clinical translation requires comparison at the product and trial levels, because similar material classes have been evaluated with different antigens, routes, disease settings, and co-therapies (Table 2C). Intramuscular V940 provides randomized evidence for adding an individualized mRNA–LNP vaccine to pembrolizumab after melanoma resection [2,85]. Intravenous autogene cevumeran provides longitudinal evidence of persistent neoantigen-specific CD8^+^ T cells in pancreatic cancer [3]. Its association between immune response and recurrence arose within a small treated cohort receiving checkpoint blockade and chemotherapy. The first study evaluates the added effect of the complete vaccine product; the second relates an immune response to subsequent clinical outcome. Neither comparison isolates a lipid variable or establishes an advantage of one administration route.

**Table 2C:** Selected clinical studies of antigen-delivery products and their translational constraints.

Product, antigen format and route	Reported study and co-therapy	Main reported finding	Delivery or manufacturing interpretation
V940/intismeran autogene: individualized mRNA in LNPs; intramuscular [2,85]	KEYNOTE-942, phase IIb; resected stage IIIB–IV melanoma; randomized 107:50 to vaccine plus pembrolizumab or pembrolizumab; NCT03897881	Primary RFS HR 0.561 (95% CI 0.309–1.017); two-sided P = 0.053, meeting the prespecified one-sided criterion. Five-year analyses were descriptive [2,85].	Design succeeded in 156/185 tissue-assessed patients; downstream manufacture in 104/105 combination-arm patients with successful design [85]. These are conditional rates. Randomization tests addition of the whole vaccine, not a lipid variable.
Autogene cevumeran: individualized mRNA–lipoplex; intravenous [3]	Phase I; resected pancreatic ductal adenocarcinoma; 16 vaccinated patients received atezolizumab; 15/16 subsequently received mFOLFIRINOX; NCT04161755	Eight of 16 patients developed the prespecified high-magnitude neoantigen-specific T-cell response; vaccine-induced CD8^+^ clones persisted approximately three years in responding patients [3].	Documents repeated intravenous vaccination and durable antigen-specific immunity. The responder/non-responder RFS association is within one treated cohort; it cannot establish treatment efficacy or superiority over intramuscular LNPs.
NeoVax: personalized synthetic long peptides with poly-ICLC; intradermal plus subcutaneous [4]	Phase I; nine patients with completely resected high-risk clear-cell renal carcinoma; five also received locally administered ipilimumab; NCT02950766	Vaccine-reactive T cells in 9/9 and autologous-tumor reactivity in 7/9; no recurrence at median 40.2 months after surgery [4].	Vaccines were manufactured and administered for all nine analyzed patients. Peptide identity, solubility and pooling remained product-specific requirements. Small, nonrandomized cohorts do not isolate route, adjuvant or checkpoint effects.
PGV001: personalized synthetic long peptides with poly-ICLC and tetanus helper peptide; subcutaneous [5]	Phase I; urothelial carcinoma; four adjuvant and six metastatic participants treated with vaccine plus atezolizumab; NCT03359239	All 10 treated participants completed priming. Objective responses occurred in 2/5 metastatic participants with measurable disease (40%; 95% CI 5.3–85.3%) [5].	Twelve participants were enrolled and 10 received vaccination. Median enrollment-to-vaccine-development time was 20.3 weeks (range 17.1–23.4), a scheduling interval rather than isolated manufacturing time [5]. Mixed disease settings, small denominators and no concurrent control restrict efficacy interpretation.
ELI-002 2P: shared mutant-KRAS peptide and CpG amphiphiles designed for albumin-mediated lymph-node delivery; subcutaneous [107,108]	AMPLIFY-201, phase I; 25 patients (20 pancreatic, five colorectal) with molecular or biomarker residual disease after locoregional treatment; NCT04853017	Initial mutant-KRAS-specific T-cell responses in 21/25 participants. Associations between immune responses and subsequent outcomes remained exploratory in the nonrandomized follow-up [107,108].	Shared antigens avoid individualized antigen selection, while restricting eligibility to the corresponding tumor mutations. Human immune responses do not directly establish albumin-mediated transport or its causal contribution to clinical benefit.
GNOS-PV02: individualized plasmid DNA; intradermal delivery with electroporation [1]	Phase I/II; 36 patients with advanced hepatocellular carcinoma after multityrosine kinase inhibitor treatment; plasmid IL-12 and pembrolizumab; NCT04251117	Neoantigen-specific T-cell responses in 19/22 evaluable patients. Investigator-assessed objective responses (confirmed plus unconfirmed) occurred in 11/36 (30.6%), including three complete responses [1].	Adds a delivery-device requirement. Vaccines were available for all 36 patients when eligible for second-line treatment; this does not define end-to-end access or a common turnaround interval. The single-arm regimen cannot isolate DNA delivery, IL-12, or checkpoint effects.
IFN-γ-Dex: autologous dendritic-cell exosomes carrying MHC-restricted tumor-antigen peptides; intradermal [89]	Single-arm phase II; 22 treated patients with advanced NSCLC without progression after platinum chemotherapy; preceding metronomic cyclophosphamide; NCT01159288	Four-month PFS was 32% (95% CI 16–53%), below the 50% target; no RECIST objective response [89].	Thirty-two of 36 preparations passed release criteria; this is a batch rate, not patient access. The missed endpoint and absence of clear adaptive immune induction prevent generalizing vesicle identity to antigen-delivery efficacy.

*Note:* Trial phases, cohort sizes, and outcomes refer to the cited publications; this selected comparison is not a census of current development programs. Trial identifiers link each product to its protocol, whose current enrollment may differ from the published analysis population. Formulations, antigen targets, disease settings, co-therapies, and endpoints differ, so the reported effects are not head-to-head platform comparisons. In KEYNOTE-942, the primary analysis met the prespecified one-sided α = 0.10 criterion [2].

*Abbreviations:* CI, confidence interval; HR, hazard ratio; IL-12, interleukin-12; LNP, lipid nanoparticle; MHC, major histocompatibility complex; NSCLC, non-small-cell lung cancer; PFS, progression-free survival; RFS, recurrence-free survival; poly-ICLC, polyinosinic–polycytidylic acid stabilized with poly-L-lysine and carboxymethylcellulose; mFOLFIRINOX, modified fluorouracil, leucovorin, irinotecan, and oxaliplatin regimen.

Patient-specific peptide vaccines expose the importance of antigen relevance and production time. In resected renal carcinoma, vaccine-induced T cells recognized autologous tumor in seven of nine participants, extending the evidence beyond peptide restimulation alone [4]. PGV001 enrolled 12 participants and vaccinated 10; median enrollment-to-vaccine-development time was 20.3 weeks [5]. This interval includes more than manufacturing alone. Peptide vaccines do not require intracellular antigen expression, but they still face sequence-specific synthesis and release requirements and coordination of vaccination with systemic therapy.

ELI-002 2P and GNOS-PV02 extend the comparison to endogenous-carrier recruitment and device-assisted delivery. ELI-002 2P uses shared mutant-KRAS peptide and CpG amphiphiles designed to bind endogenous albumin; its phase I trial and follow-up document antigen-specific immunity in a non-individualized vaccine approach [107,108]. Eligibility still depends on tumor antigen status, and the clinical studies do not directly isolate albumin-mediated transport. GNOS-PV02 combines individualized plasmid DNA with electroporation, plasmid interleukin-12 (IL-12), and pembrolizumab [1]. Its clinical activity supports evaluation of the combined regimen while leaving the separate contributions of DNA delivery, IL-12, and checkpoint blockade unresolved.

Manufacturing success, receipt of treatment, antigen-specific function, and clinical benefit also require separate denominators. V940 manufacturing success was reported conditional on successful vaccine design [85]. Dendritic-cell-exosome preparations could meet release criteria while the non-small-cell lung cancer (NSCLC) trial missed its progression-free-survival target and showed no objective response [89]. A useful clinical comparison identifies the barrier each product addressed and the evidence still needed. Differences in cancer type, disease burden, antigen selection, and accompanying therapy prevent these studies from establishing a hierarchy of carrier efficacy.

Later development outcomes require separate interpretation. In sponsor-reported results dated 15 June 2026, the randomized phase II AMPLIFY-7P trial of ELI-002 7P missed its prespecified disease-free-survival endpoint in the intention-to-treat population; subgroup and immune-response associations remain exploratory [109]. This trial tested a different formulation from the published ELI-002 2P cohort. On 28 August 2026, BioNTech announced termination of autogene cevumeran monotherapy in resected colorectal cancer following a monitoring committee recommendation, citing low likelihood of efficacy and a numerical overall-survival imbalance. No new safety signal was reported, and the separate pancreatic-cancer phase II combination trial was unaffected [110]. These sponsor disclosures update program status without establishing a class-wide carrier effect.

## 3 Interface engineering and signal co-localization

Surface and interface variables fall into two distinct axes (Fig. 2). The target cell–receptor axis determines which cell subset and uptake pathway a locally available carrier engages, whereas the anatomical interface axis determines whether the carrier reaches and persists at the required tissue. Because one modification can affect both axes, a targeting claim must identify which axis the modification affects and assess the other as a potential confounder.

**Fig. 2:**
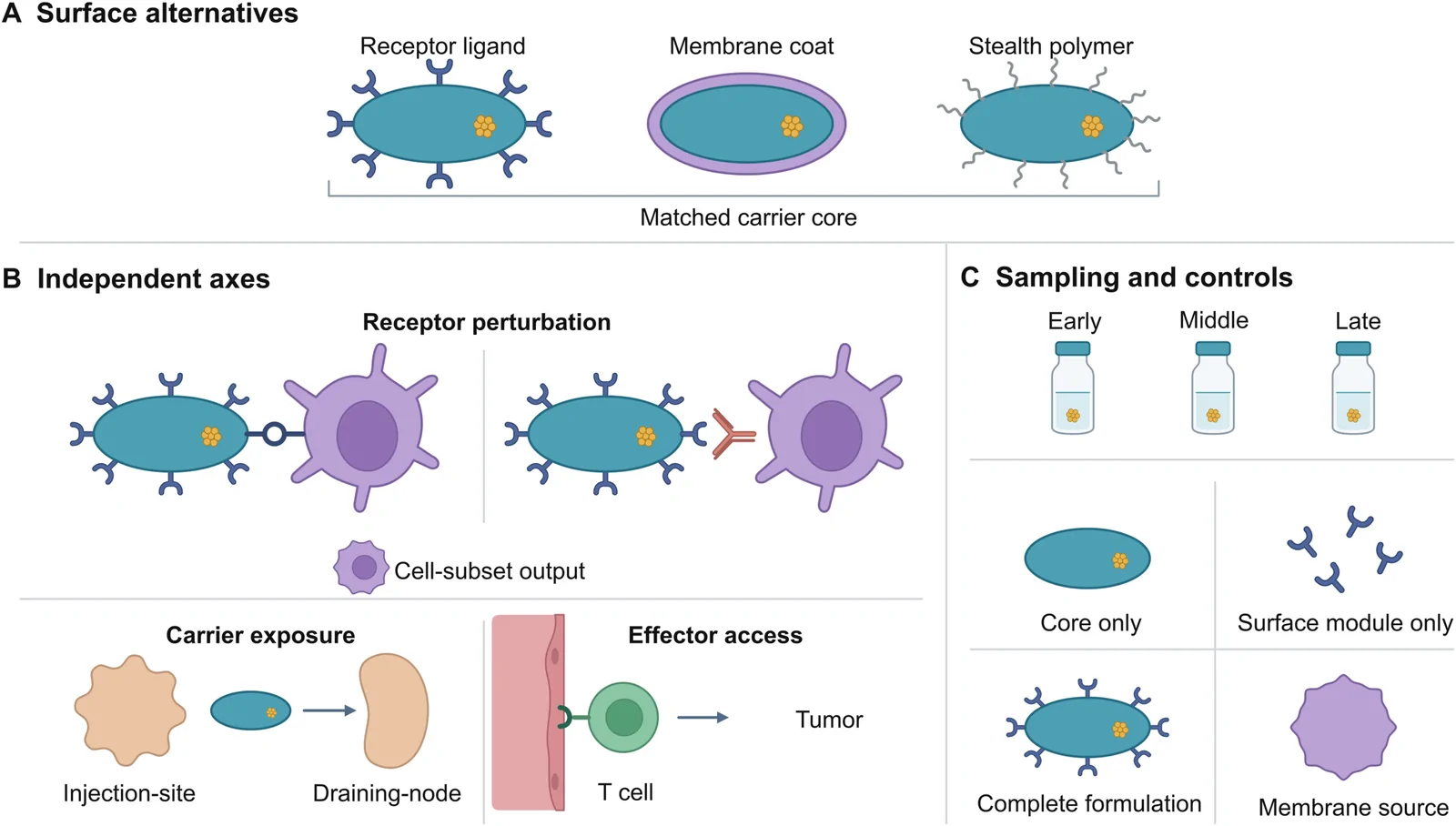
Surface alternatives, independent biological axes, and matched sampling controls. (A) Receptor ligand, membrane coat, and stealth polymer are shown as alternative surface designs on a matched carrier core; they are not assumed to be combined. (B) Independent axes distinguish receptor perturbation and cell-subset output from carrier exposure between the injection site and draining node and from T-cell effector access to tumor. (C) Early, middle, and late sampling is paired with core-only, surface-module-only, complete-formulation, and membrane-source controls. The receptor-related cellular-output and anatomical-exposure axes do not form a serial mechanistic chain. Panels A–C are qualitative and not drawn to scale. Created in BioRender. Wang, S. (2026) https://BioRender.com/1meeco7.

### 3.1 Engineering the target cell–receptor axis

The target cell–receptor axis connects a surface variable to receptor-dependent uptake in a prespecified cell subset, followed by intracellular routing, peptide–major histocompatibility complex (pMHC) formation and surface display, and antigen-specific function. Aptamer-functionalized particles and native scaffold–receptor interactions test cell-subset entry, whereas source-derived membranes provide a broader interface that can affect both exposure and uptake [39,67,68,111,112]. None specifies a preferred cell or site without context-specific receptor and distribution data. Increased association is insufficient when receptor dependence is not demonstrated or uptake terminates in degradation. Causal attribution requires carriers matched for antigen dose, size, charge, and adjuvant exposure. Receptor perturbation and cell-subset-resolved uptake can then distinguish receptor-dependent targeting from receptor-independent or nonspecific uptake, while pMHC and T-cell measurements determine whether the altered uptake is productive [113,114].

The 24-subunit heavy-chain ferritin (H-ferritin) cage binds transferrin receptor 1 (TfR1) and undergoes receptor-mediated uptake [111,112]. TfR1 is also expressed by normal proliferating cells and erythroid-lineage cells, so receptor expression does not confer tumor specificity. In this context, H-ferritin provides a receptor-mediated uptake variable, whereas valency, fusion geometry, and assembly state are separate architectural variables. A vaccine-delivery gain can be attributed to TfR1 engagement only when receptor-dependent uptake occurs in the intended APC subset and is followed by increased surface pMHC abundance and antigen-specific function.

### 3.2 Engineering the anatomical interface

The anatomical interface axis links a formulation or source-derived interface to exposure at the injection site, draining lymph node, or tumor and then to encounter with the intended cell type and an antigen-specific outcome. Size, charge, PEG content, hydrophobicity, and ligand density can alter drainage, retention, and interstitial distribution [33,59,60]. A single terminal time point cannot establish exposure over time and space. Lymph-node accumulation is informative only with uptake by the relevant APC subset and priming; tumor retention likewise requires local antigen presentation or effector access.

Biomimetic membranes and EVs add a source-derived interface whose adhesion proteins, endogenous cargo, and innate cues can alter trafficking and immune recognition [39,40,[68], [69], [70]]. Engineered EV systems can incorporate peptide or DNA cargo [115,116], while exosome-based and outer membrane vesicle–based systems extend this principle [117,118]. Source-cell state, isolation, storage, co-isolated material, and surface composition may change together, so attribution requires configuration-appropriate core-only, surface-module-only, complete-formulation, or membrane-source controls (Fig. 2C). Immune checkpoint ligands modify immune context but do not establish initial cell or tissue exposure. The receptor-related cellular-output examples in Fig. 3A–C and the anatomical-exposure examples in Fig. 3D–G are therefore shown as independent study blocks rather than one causal sequence.

**Fig. 3:**
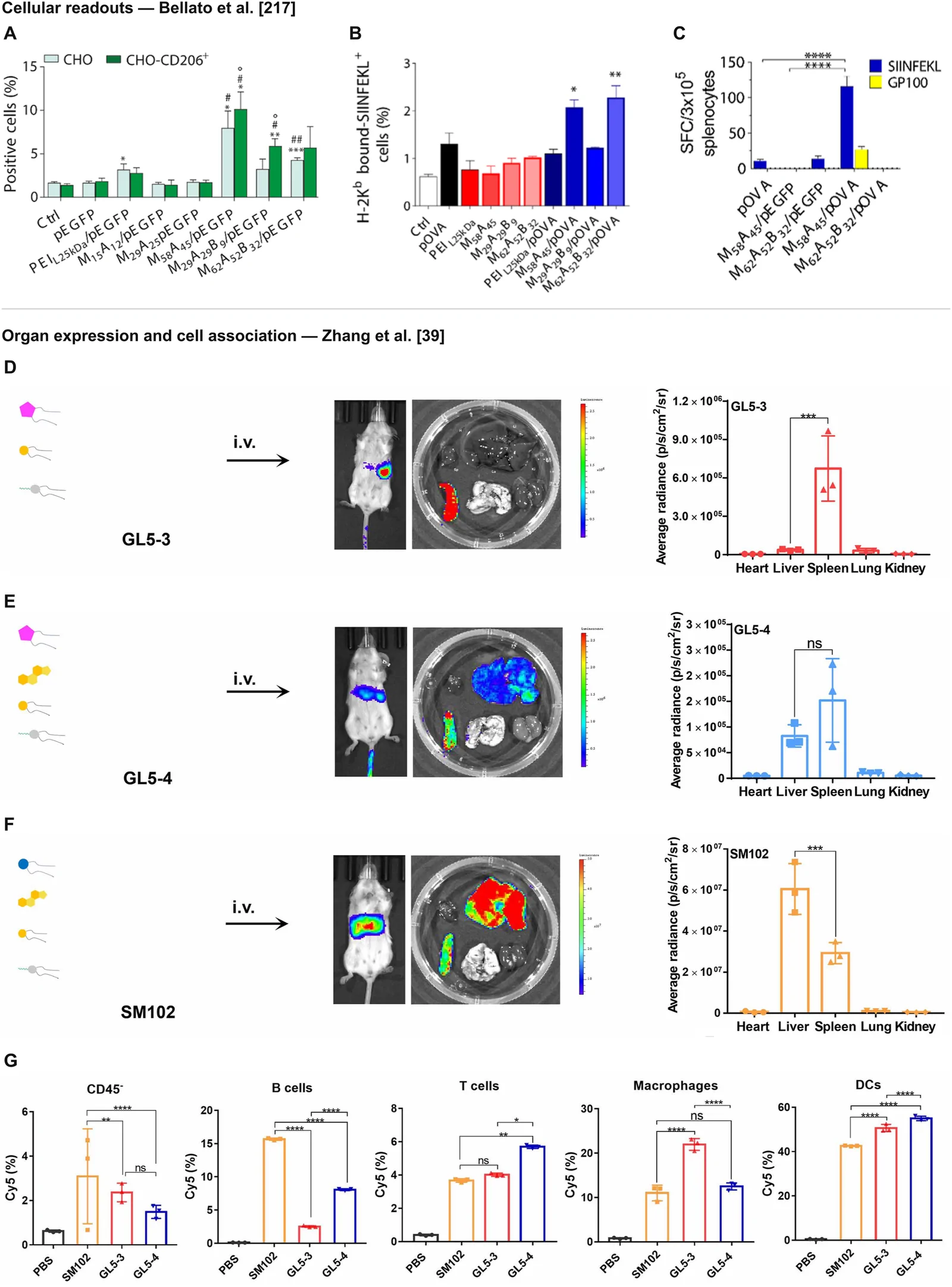
Distinct experimental readouts for receptor-related cellular outputs and anatomical exposure. (A–C) Cellular readouts from Bellato et al. [119]. (A) Percentage of EGFP-positive CHO and CHO-CD206^+^ cells after treatment with the indicated pEGFP formulations; this is a transfection-output readout, not a measure of particle uptake. (B) Percentage of JAWS II cells positive for surface H-2K^b^–SIINFEKL after treatment with the indicated pOVA formulations. (C) Interferon-γ (IFN-γ) enzyme-linked immunosorbent spot responses of splenic T cells from B16-OVA-bearing mice after restimulation with SIINFEKL or gp100. These panels use different cargos, models, and assays and therefore do not form a serial mechanistic chain. Panels A–C are adapted from source Fig. 3a, 5c and 6f in Bellato F, Feola S, Dalla Verde G et al., “Mannosylated polycations target CD206^+^ antigen-presenting cells and mediate T-cell-specific activation in cancer vaccination,” *Biomacromolecules* 23, 5148–5163 (2022), https://doi.org/10.1021/acs.biomac.2c00993, under the Creative Commons Attribution 4.0 International License (https://creativecommons.org/licenses/by/4.0/). (D–G) Organ reporter expression and cell association from Zhang et al. [55]. (D–F) GL5-3, GL5-4, and SM102 lipid nanoparticle designs, respectively, with whole-body and ex vivo organ bioluminescence and average organ radiance after intravenous delivery of luciferase mRNA. (G) Percentages of Cy5-positive CD45^−^ cells, B cells, T cells, macrophages, and dendritic cells in the spleen after exposure to G-LNPs or SM102 LNPs. These panels report organ-level reporter expression and fluorescent-label positivity or cell association; they do not by themselves establish productive antigen presentation or antigen-specific function. Panels D–G are adapted from source Fig. 5a–c and 6a in Zhang H, Liu D, Yang K, Liang Z, and Li M, “Ionizable guanidine-based lipid nanoparticle for targeted mRNA delivery and cancer immunotherapy,” *Science Advances* 11, eadx5970 (2025), https://doi.org/10.1126/sciadv.adx5970, under the Creative Commons Attribution 4.0 International License (https://creativecommons.org/licenses/by/4.0/). The Bellato and Zhang panels derive from independent studies and do not form a cross-study causal sequence.

### 3.3 The tumor microenvironment as a second delivery-to-immunity bottleneck

Carrier exposure and APC uptake address delivery steps upstream of priming, but tumor access remains a separable downstream bottleneck. A carrier may improve delivery to lymph-node APCs and expand antigen-specific T cells while leaving endothelial entry, movement through collagen-rich stroma, proximity to tumor cells, and function within suppressive myeloid or metabolic niches unchanged [[12], [13], [14]]. In human urothelial tumors, transforming growth factor-β signaling in fibroblasts was associated with exclusion of CD8^+^ T cells from tumor parenchyma [120]. Dynamic imaging of viable human lung tumor slices linked macrophage-mediated trapping to reduced CD8^+^ T-cell motility and access to tumor islets [121]. “Second delivery-to-immunity bottleneck” denotes access and function of vaccine-elicited effector cells, not delivery of the antigen carrier.

For a lymph-node-directed platform, evidence must connect APC encounter with presentation and priming. Tumor-directed or *in situ* systems must also show effector infiltration or tumor-cell proximity and retained function. If priming persists without intratumoral access or activity, the unresolved bottleneck is effector-cell access or function within the tumor microenvironment, not antigen delivery to APCs. Tumor control alone cannot identify which bottleneck was resolved.

Reaching a target site does not ensure that antigen and an immune signal coincide. Physical co-delivery means that both components share one particle; same-tissue modulation is a tissue-level co-localization configuration without requiring a shared carrier; systemic co-administration combines separate treatments at the regimen level. Each configuration requires claim-matched evidence and should not be relabelled as another category (Fig. 4A–C).

**Fig. 4:**
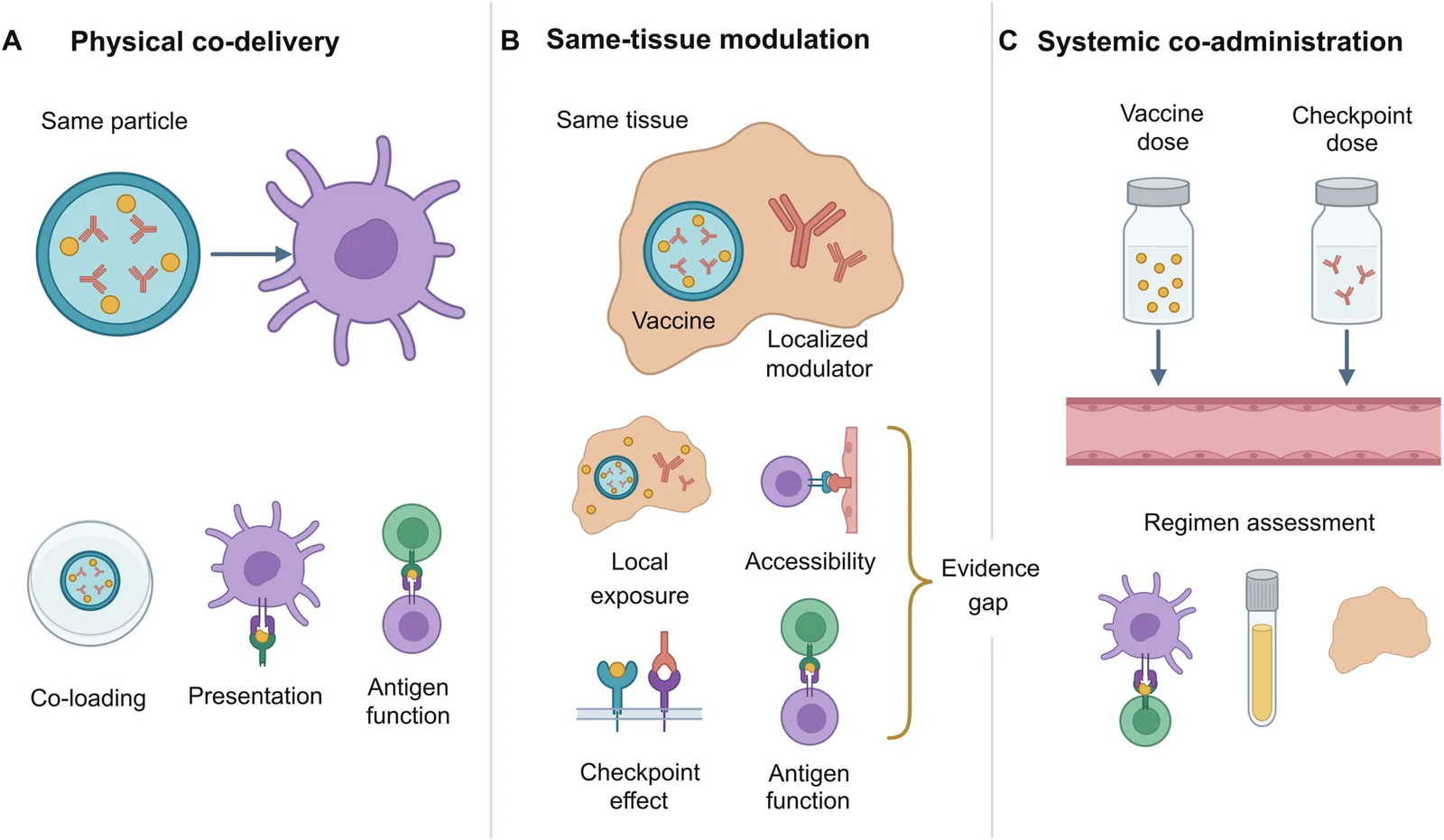
Physical co-delivery, same-tissue modulation, and systemic co-administration define distinct combination configurations. (A) Physical co-delivery places antigen and an immune-modulatory signal in the same particle; co-loading, presentation, and antigen-specific function remain separate measurements. (B) Same-tissue modulation keeps the vaccine and localized modulator physically separate and retains local exposure, accessibility, checkpoint effect, and antigen-specific function as an explicit evidence gap rather than an assumed sequence. (C) Systemic co-administration uses separate vaccine and checkpoint doses and is evaluated at the regimen level. These configurations are not ranked. Regimen-level co-administration does not establish physical co-delivery; attribution of individual component effects requires appropriate comparator arms. Panels A–C are qualitative and not drawn to scale. Created in BioRender. Wang, S. (2026) https://BioRender.com/1meeco7.

### 3.4 Antigen–adjuvant physical co-delivery and co-localization

Peptide conjugates bearing Toll-like receptor 7/8 (TLR7/8) agonists, manganese dioxide (MnO_2_) nanoparticles, and protein-delivering dendrimers couple antigen uptake with APC activation [38,46,77]. The benefit of same-particle physical co-delivery depends on dose ratio, release order, and intracellular destination. A co-localized nanomedicine–vaccine regimen acts at the tissue level [78].

Endosomolytic polymersomes do not provide physical co-delivery evidence because the cited study tested intracellular routing of a stimulator of interferon genes (STING) agonist without antigen co-loading [86]. Verified co-loading establishes the physical configuration (Fig. 4A). Demonstrating an advantage of that configuration additionally requires cellular co-uptake or tissue co-distribution to be linked to pMHC presentation and antigen-specific T-cell responses.

An antigen–adjuvant dose matrix with vehicle-, antigen-, and adjuvant-only controls is needed to distinguish co-localization from dose escalation or parallel activation. Formulation characterization and pharmacokinetic (PK) measurements can then be related to co-distribution, release order, presentation, T-cell function, and toxicity.

### 3.5 mRNA vaccination and immune checkpoint modulation

mRNA must remain protected long enough to enter a suitable APC and support antigen expression, after which the antigen must be processed into surface pMHC complexes [122,123]; expression alone does not establish vaccine function. Protein-scaffold and shared-antigen strategies provide comparators to individualized mRNA formats [98,124], while sequence changes make production time a product-level constraint [81]. These format and production constraints define the vaccine component, whereas the combination classification depends on how immune checkpoint modulation is delivered.

Systemic programmed cell death protein 1 (PD-1) or programmed death ligand 1 (PD-L1) inhibition is regimen-level co-administration, not physical co-delivery [1,2,5,123]. A surface-bound or locally released immune checkpoint modulator, such as a material functionalized with a PD-L1-targeting aptamer, instead represents same-tissue modulation [67] (Fig. 4B). A shared carrier requires verified co-loading and co-distribution, compatible release, and preserved component activity. The preclinical and human regimen examples in Fig. S1A–C[125] and S1D–E remain independent and do not establish physical co-delivery. The preclinical controls permit assessment of component effects on a common adjuvant background, whereas the single-arm human study does not isolate the vaccine contribution.

Clinical results caution against inferring efficacy from mechanistic compatibility. In KEYNOTE-942, 157 patients were randomized 107:50 to V940 plus pembrolizumab or pembrolizumab. The primary recurrence-free survival analysis was positive under the prespecified protocol definition with an overall one-sided alpha of 0.10 (hazard ratio, 0.561; 95% confidence interval, 0.309–1.017); the reported two-sided *P* value was 0.053 [2]. In the 5-year update (median planned follow-up, 60.3 months), updated recurrence-free survival and distant metastasis-free survival hazard ratios were 0.510 and 0.411, whereas overall survival data remained immature (hazard ratio, 0.471; 95% confidence interval, 0.165–1.345). These analyses were descriptive with no allocated alpha [85].

On 19 August 2026, the sponsors announced that phase III INTerpath-001 met its primary recurrence-free-survival and key secondary distant-metastasis-free-survival endpoints [84]. The announcement did not disclose phase III effect estimates; the hazard ratios above belong to the phase IIb study. In a 692-patient phase 3 trial, talimogene laherparepvec (T-VEC) plus pembrolizumab did not improve progression-free survival (hazard ratio, 0.86; *P* = 0.13) or overall survival (hazard ratio, 0.96; *P* = 0.74) [126]. Neither regimen tests physical co-delivery or isolates a carrier variable.

### 3.6 Local cytokine, myeloid, and metabolic modulation

When linked to vaccination, local interleukin delivery, transforming growth factor-β blockade, myeloid-cell modulation, and hypoxia-responsive modules may address post-priming barriers [[12], [13], [14]]. An *in situ* hydrogel that alters antigen presentation by type 1 conventional dendritic cells provides one example in a vaccine context [127]. Reviews of nanoadjuvants, postoperative residual disease, radiotherapy, and organ-specific delivery provide context [[128], [129], [130], [131]], but not direct therapeutic-vaccine evidence.

Systemic or unencapsulated controls are needed to distinguish localization from modulator pharmacology. Cytokine abundance alone is insufficient; local exposure and myeloid-cell phenotype or function must be interpreted alongside antigen-specific T-cell function, tumor infiltration, and tolerability. An effect retained in an antigen-free control may instead reflect local immunotherapy, so attribution should be judged against configuration-specific evidence (Table 3).

**Table 3:** Delivery configurations and evidence needed to distinguish physical co-delivery, tissue-level co-localization, and regimen-level co-administration.

Configuration	Biological relationship	Evidence for attribution	Principal alternative explanation
Antigen plus Toll-like receptor agonist in one carrier [46]	Physical co-delivery intended to coordinate same-APC exposure and activation	Co-loading, cellular co-uptake, release sequence, surface pMHC abundance, antigen-specific T cells	Dose escalation, systemic inflammation, tolerogenic routing
Antigen plus STING agonist in one carrier [36]	Physical co-delivery intended to couple antigen processing with intracellular STING signaling	Verified co-loading and intracellular delivery; pMHC and antigen-specific T-cell response	Agonist routing demonstrated without antigen co-delivery [86]
Vaccine plus systemic immune checkpoint inhibitor [2]	Regimen-level co-administration without physical co-delivery	Antigen-specific vaccine response, schedule, systemic safety, and clinical endpoint	Co-administration mislabeled as co-delivery
Vaccine plus locally retained or released immune checkpoint modulator [67]	Tissue-level co-localization without requiring a shared carrier	Tissue localization, modulator accessibility or release, local immune checkpoint effect, antigen-specific function	Insufficient local concentration or carrier-driven effect
Vaccine and immune checkpoint modulator in the same carrier	Physical co-delivery (conceptual configuration; design context [35])	Co-loading, PK/co-distribution, independent component function, and matched single-component controls	Incompatible dose, loading, or release requirements
Antigen plus a cytokine or a modulator targeting myeloid cells or metabolism [12,127]	Tissue-level co-localization targeting a post-priming barrier	Local exposure, antigen-specific response, relevant myeloid or metabolic change, tolerability	Generalized inflammation or non-vaccine antitumor activity

*Note:* References provide representative studies or design context; attribution criteria and alternative explanations are this review's synthesis. Reference [67] concerns local photoimmunotherapy, not selected-antigen co-loading. The shared-carrier checkpoint row is a conceptual configuration, with [35] providing design context rather than direct evidence for that combination.

*Abbreviations:* APC, antigen-presenting cell; PK, pharmacokinetics; pMHC, peptide–major histocompatibility complex; STING, stimulator of interferon genes.

## 4 Release, intracellular routing, and antigen architecture

Verified co-delivery or co-localization does not establish productive antigen presentation. Once a formulation reaches the intended APC, its antigen may still be released extracellularly before uptake, remain trapped in the carrier, undergo lysosomal degradation, or separate from its immunomodulatory signal [[9], [10], [11]]. The route must therefore be tested as separate inference steps: conditional release is not *in vivo* delivery, uptake is not surface pMHC, and pMHC display is not antigen recognition (Fig. 5A–C).

**Fig. 5:**
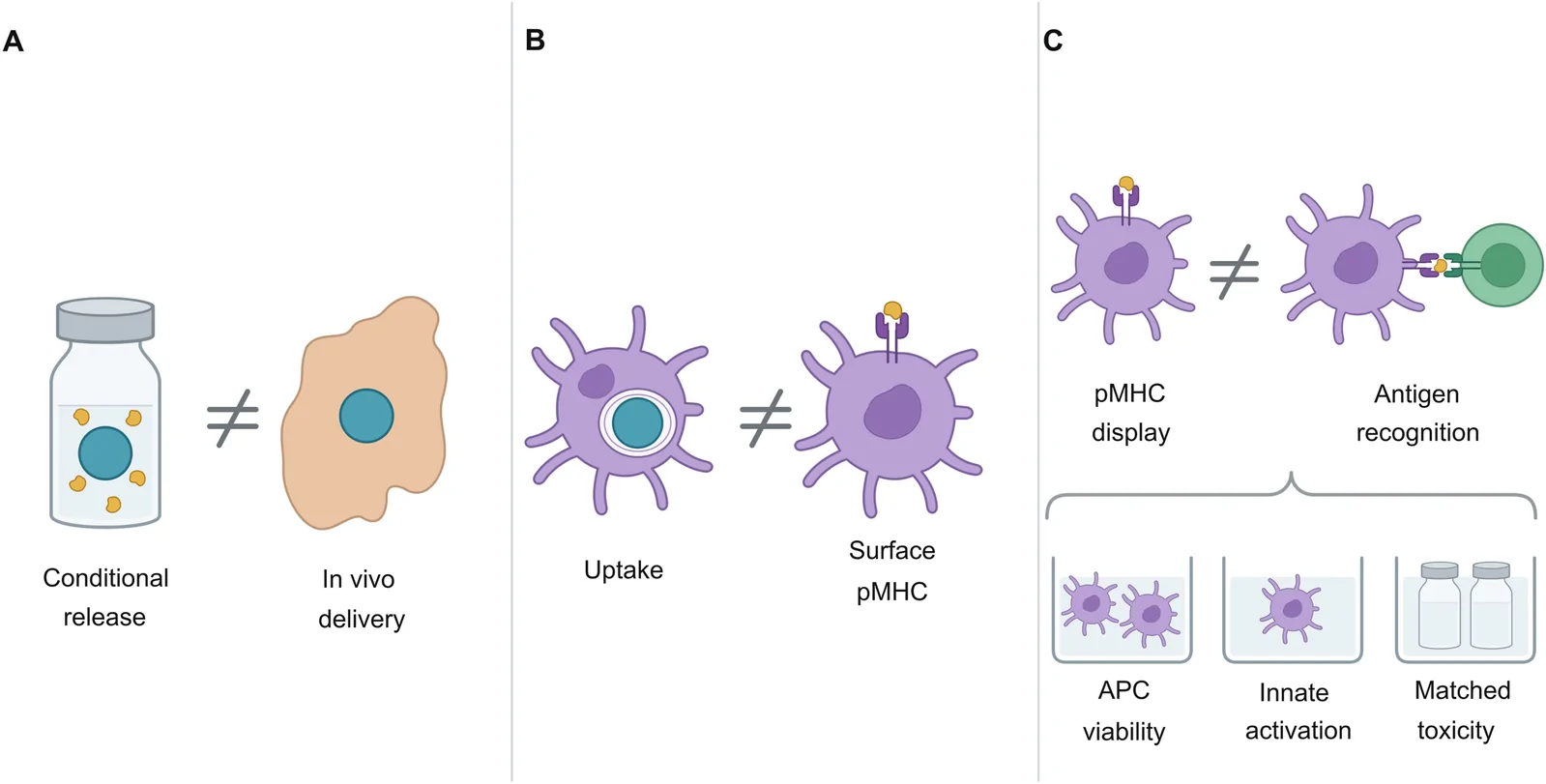
Release, uptake, surface pMHC display, and antigen recognition are non-equivalent evidence layers. (A) Condition-dependent release does not establish delivery *in vivo*. (B) Cellular uptake does not establish surface peptide–major histocompatibility complex (pMHC) display. (C) Surface pMHC display does not itself establish antigen-specific recognition; parallel assessments of antigen-presenting-cell viability, nonspecific innate activation, and dose-matched toxicity are also needed. These evidence layers do not establish a universal intracellular mechanism. Created in BioRender. Wang, S. (2026) https://BioRender.com/1meeco7.

**Fig. 6:**
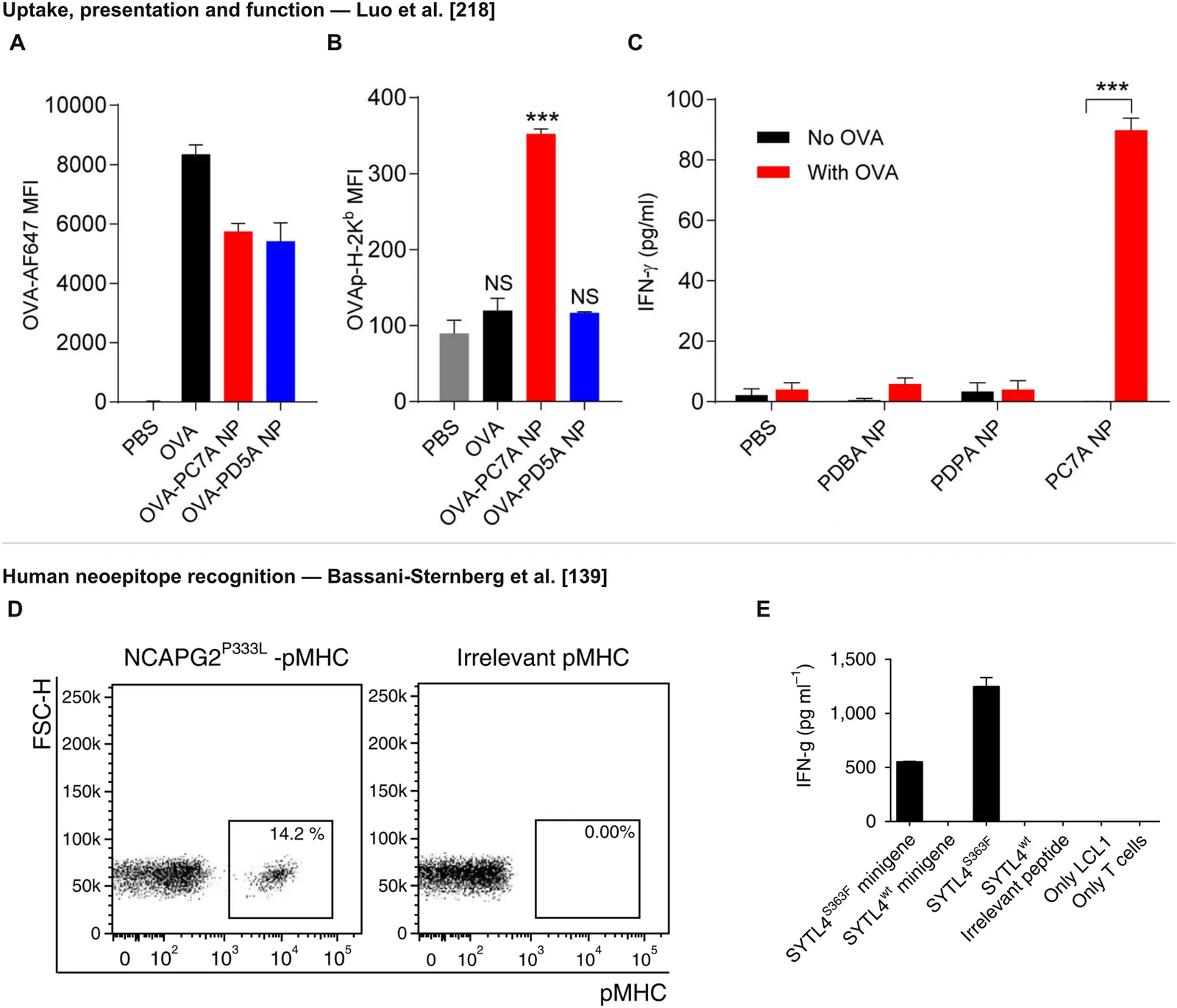
Uptake, surface presentation, and antigen-specific recognition are distinct readouts. (A–C) Uptake, presentation, and antigen-specific function from Luo et al. [132]. (A) Mean fluorescence intensity of AF647-OVA in bone-marrow-derived dendritic cells (BMDCs) after 4 h incubation with AF647-OVA alone, AF647-OVA-PC7A nanoparticles, or AF647-OVA-PD5A nanoparticles. (B) Surface OVA–H-2K^b^ presentation in BMDCs after treatment with the indicated OVA formulations. (C) IFN-γ secretion by OT-I CD8^+^ T cells after coculture with BMDCs treated with the indicated OVA formulations. These same-study endpoints distinguish uptake, surface peptide–major histocompatibility complex (pMHC) display, and antigen-specific function, but they do not directly measure cytosolic localization or establish a universal endosomal-escape mechanism. Panels A–C reproduce source Fig. 2c–e from Luo M, Wang H, Wang Z et al., “A STING-activating nanovaccine for cancer immunotherapy,” *Nature Nanotechnology* 12, 648–654 (2017), https://doi.org/10.1038/nnano.2017.52, © 2017 Springer Nature Limited, reproduced with permission from Springer Nature Customer Service Centre GmbH under License No. 6330861438112. This material is not included in the Creative Commons licence governing the present article; further reuse requires permission from the rights holder. (D–E) Human neoepitope recognition from Bassani-Sternberg et al. [133]. (D) Staining of a Mel15-derived NCAPG2^P333L^-specific T-cell line with the specific pMHC multimer compared with an irrelevant multimer. (E) IFN-γ secretion by a SYTL4^S363F^-specific T-cell clone after coculture with peptide-pulsed or minigene-transduced LCL1 target cells. Panels D and E use different neoepitopes and T-cell preparations and are therefore independent recognition examples rather than a D-to-E causal sequence. The study establishes recognition of naturally presented human melanoma neoepitopes but is not a delivery-platform experiment and does not show that a material increased presentation. Panels D–E are adapted from source Fig. 5g and h in Bassani-Sternberg M, Bräunlein E, Klar R et al., “Direct identification of clinically relevant neoepitopes presented on native human melanoma tissue by mass spectrometry,” *Nature Communications* 7, 13404 (2016), https://doi.org/10.1038/ncomms13404, under the Creative Commons Attribution 4.0 International License (https://creativecommons.org/licenses/by/4.0/). The Luo and Bassani-Sternberg panels derive from independent studies, address only uptake, presentation, or recognition, and do not form a causal sequence; none supplies release evidence.

### 4.1 Environment-responsive release: evidence and limits

Carrier-free vaccines, manganese-coordinated particles, biomineralized systems, and hydrogels use responsive chemistry to alter cargo availability [36,62,63,127]. A buffered release curve does not establish delivery *in vivo*: tumor interstitium, endosomes, and lysosomes differ in pH and enzymes across cells and tumors. A single-buffer threshold can therefore overstate selectivity or miss premature serum release (Fig. 5A).

Timing is equally important. Antigen released before APC maturation may lack co-stimulation, whereas adjuvant released alone may cause inflammation without antigen-specific priming. Sustained exposure can also desynchronize antigen and danger signals. Relevant-compartment assays should therefore connect serum stability and trafficking to pMHC presentation and T-cell responses.

### 4.2 Endosomal escape, cytosolic transfer, and cross-presentation

For vaccines intended to prime CD8^+^ T cells, uptake must be distinguished from productive MHC-I antigen presentation [[9], [10], [11],^113^,^114^]. As a routing comparator, endosomolytic polymersomes increased 2′3′-cyclic GMP–AMP (cGAMP) uptake and STING activity; the authors inferred that endosomal escape and cytosolic delivery accounted for much of the potency gain, but cytosolic cGAMP was not measured directly [86]. Protein nanoparticles can alter lymph-node access, APC uptake, and processing [98,134]. Neither observation proves that every membrane-disruptive or particulate formulation improves antigen cross-presentation.

Increasing membrane disruption is not necessarily beneficial. Although it can increase cytosolic transfer, it can also injure APCs, trigger nonspecific inflammation, release cargo prematurely, or alter uptake. At a matched dose, subcellular localization and surface pMHC display should therefore be interpreted together with APC viability, innate immune activation, matched toxicity controls, and antigen-specific T-cell function (Fig. 5B and C). Panels 6A–C provide same-study uptake, surface pMHC presentation, and antigen-specific functional endpoints, whereas panels 6D–E provide independent natural-recognition examples; the two blocks do not define a shared intracellular route.

Release mechanisms also require scaffold-specific attribution. Studies of ferritin-based drug delivery demonstrate formulation-dependent encapsulation and release [[135], [136], [137], [138]]. A biochemical comparison reported differences in pH-dependent disassembly among ferritins of different origins and subunit compositions [139]. Stability, release, and processing must therefore be measured for the specific scaffold.

### 4.3 Externally triggered and *in situ* vaccination comparators

As boundary comparators for carrier-intrinsic release, metal–organic framework (MOF) photodynamic or photoimmunotherapy systems can couple tumor damage to immune activation [64,65,67,140]; high-intensity focused ultrasound-responsive or immune-regulating MOFs [66,141] and ferritin photoimmunotherapy [142] provide distinct contexts. Rather than delivering a preselected vaccine antigen, most make tumor-derived antigens available *in situ*. They therefore inform local antigen generation and immune activation without belonging to the same carrier class.

External triggering is limited by tissue penetration and anatomical accessibility, and its timing must be coordinated with uptake, image guidance, surgery, or radiotherapy. Tumor regression or inflammation alone cannot establish vaccine benefit. Material tracking and product characterization assess localization, trigger response, and release; immunopeptidomic methods, including major histocompatibility complex-associated peptide proteomics (MAPPs), identify presented peptides [133,143,144]; antigen-specific T-cell assays test functional relevance.

Architecture controls antigen valency (copy number), spacing, orientation, linker design, surface accessibility, and processing access (Fig. 7) [17,41,[71], [72], [73]]. Comparisons among protein cages, VLPs, peptide assemblies, and DNA origami reveal platform constraints but do not by themselves isolate geometry. In protein cages, geometry-related variables and scaffold visibility can be evaluated without implying greater clinical maturity than LNP regimens.

**Fig. 7:**
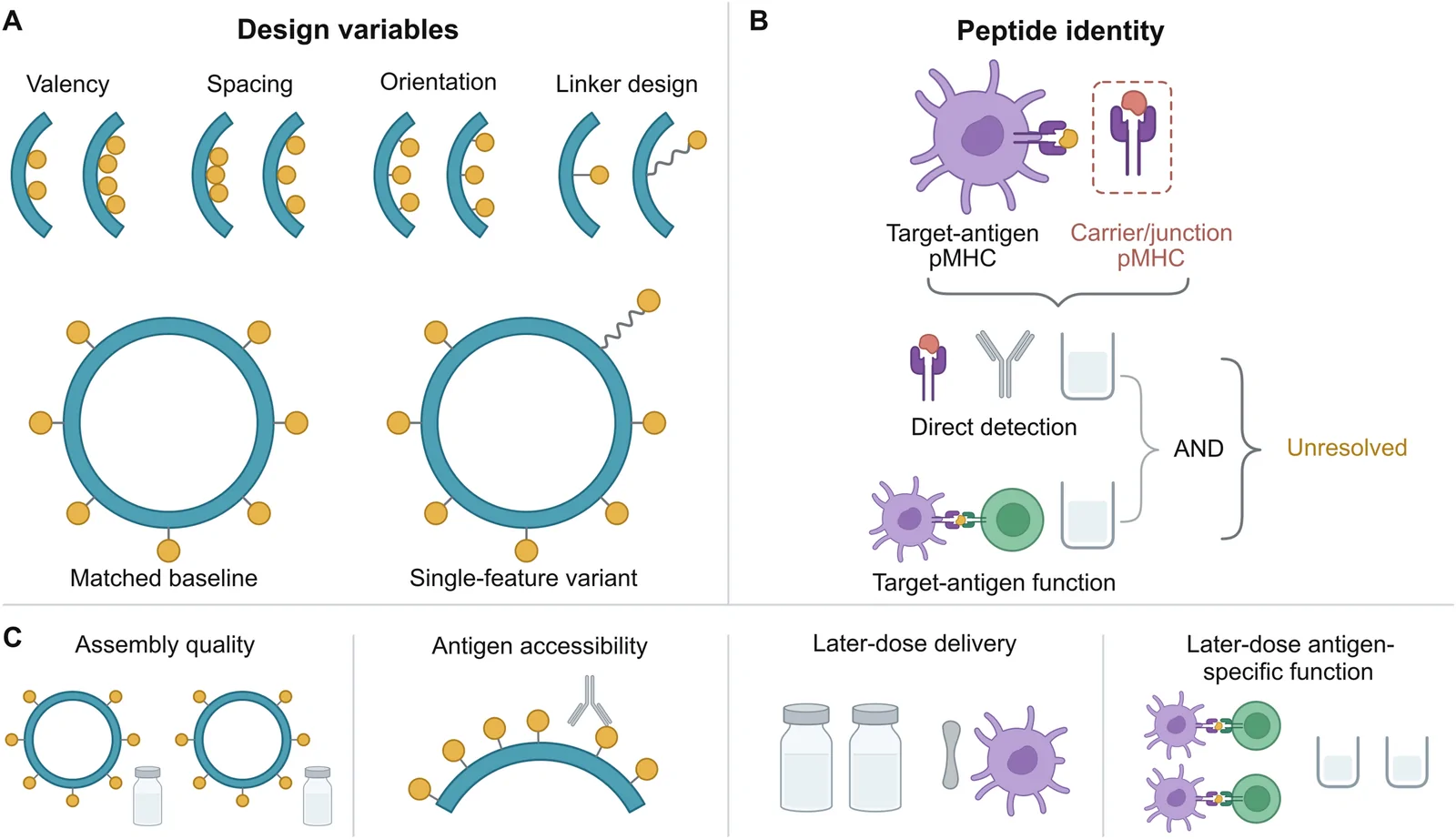
Scaffold design variables, peptide identity, and validation domains require separate evaluation. (A) Valency, spacing, orientation, and linker design are distinct scaffold variables; a matched baseline and single-feature variant enable one-variable testing where technically feasible. (B) Target-antigen pMHC is distinguished from carrier- or junction-derived pMHC. Direct detection of carrier/junction pMHC and target-antigen-specific function form a joint evidence requirement, which remains unresolved until both are measured in a claim-matched system. (C) Assembly quality, antigen accessibility, later-dose delivery, and later-dose antigen-specific function are parallel validation domains. Deposited structures and experimental-density comparisons cannot resolve carrier- or junction-derived pMHC identity or establish causal effects of scaffold variables. Panels A–C are qualitative and not drawn to scale. Created in BioRender. Wang, S. (2026) https://BioRender.com/1meeco7.

### 4.4 Geometry has immune-context-specific effects

Repetitive display can cluster B-cell receptors and alter response composition [[145], [146], [147], [148]], but preventive evidence from DNA origami or ferritin [43,134], mechanistic particle-display work [146], and VLP or vaccine precedents [[149], [150], [151]] cannot establish therapeutic cancer-vaccine antigen delivery or T-cell priming. Productive delivery still requires APC entry, antigen processing, pMHC formation, and surface display [113,114]. The human ferritin heavy chain (hFTN)-red fluorescent protein (RFP) study measured lymph-node retention and a CD8^+^ T-cell interferon-γ (IFN-γ) response after ex vivo restimulation with an RFP-derived peptide, not direct APC uptake or surface pMHC [152]. In the eOD-GT5 valency study, epitope accessibility and antigen density were not perfectly matched across constructs [145].

Causal experiments should vary valency, spacing, or orientation while matching antigen dose, adjuvant, carrier, and route, then relate surface accessibility and pMHC to antigen-specific function. Soluble antigen tests a broader particle effect. A lower-valency control tests valency, whereas a density-matched spatially disordered control tests spatial ordering; each is informative only if other relevant variables are also matched.

### 4.5 Assembly, attachment, and fusion are coupled variables

Carrier-free antigen–adjuvant assemblies have been reported [46,62]. By contrast, a dendrimer formulation is polymeric [77], whereas another regimen achieves tissue-level co-localization rather than carrier-free assembly [78]. Changing a personalized antigen may alter assembly, stoichiometry, surface accessibility, and release, so delivery must be re-established after substitution.

Protein cages and VLPs can elicit scaffold responses [90,92,95], with context-dependent effects on antigen-specific function [91,93,94]. De novo cage designs [41,[71], [72], [73]] underpin multivalent vaccine displays [[153], [154], [155], [156]]. Genetic fusion, SpyTag/SpyCatcher-mediated conjugation, and sortase-mediated ligation provide defined attachment methods [[157], [158], [159], [160]]. Coupling yield, site, orientation, and assembly require characterization. Direct evidence of junction-derived pMHC, such as sequence-resolved immunopeptidomic detection, is needed to establish presentation; a claim that such presentation alters target-antigen-specific function additionally requires functional evidence.

Ferritin examples must be identified beyond the family name. H-ferritin architecture [161], broader composition, receptor interaction, and structural variation [111,112,162,163], engineered constructs [164], and archaeal ferritin [165] carry different constraints. Ferritin scaffolds have displayed tumor antigens [98,99,152,164]. A metalloprotein *in situ* vaccine [100] and non-ferritin protein or biopolymer particles [101,166] are boundary comparators. Small-molecule ferritin studies [[135], [136], [137], [138]] inform loading, while H-ferritin drug-delivery data caution against class-wide inference [167].

### 4.6 Precision geometry as a comparator

DNA origami enables precise antigen spacing [43], whereas quasisymmetric protein cages make scaffold topology adjustable [168,169]. DNA structures can engage the cyclic GMP–AMP synthase (cGAS)–STING pathway or Toll-like receptor 9 (TLR9) signaling and face distinct stability and scale constraints [44,45]. Protein cages have established expression and assembly workflows but add questions about scaffold immunity and sequence-dependent assembly [17,91,92,96,97].

A translational architectural advantage requires matched evidence that geometry changes processing or recognition, persists during repeat dosing, and remains reproducible in manufacture. These conditions make “programmability” testable rather than an assumed property of an ordered structure (Fig. 7A and C). The deposited structure pairs in Fig. 8A and B verify distinct local linker endpoints but do not establish an immune consequence.

**Fig. 8:**
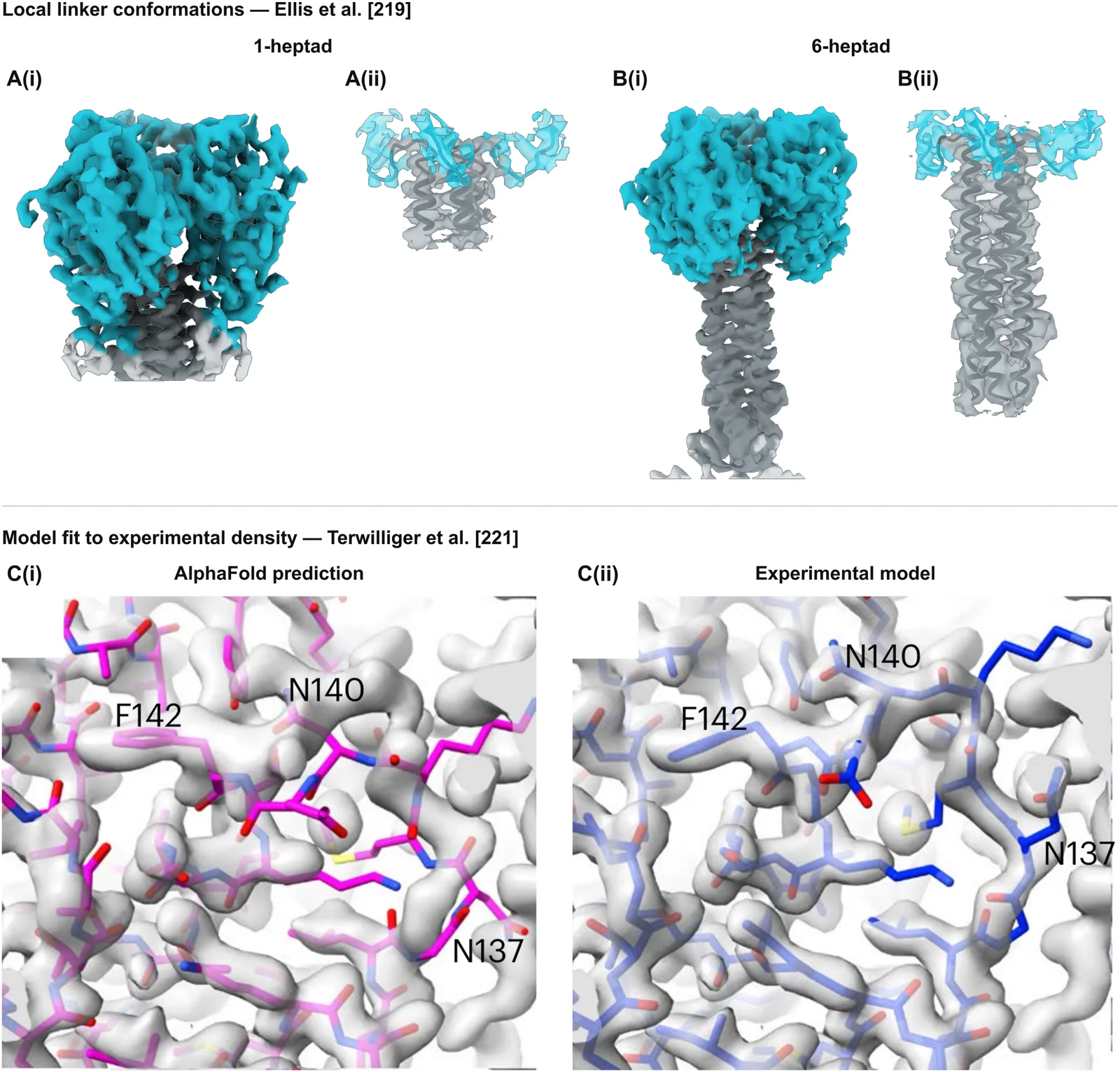
Local linker conformations in deposited structures and experimental-density assessment of model fit. (A) UCSF ChimeraX renderings of the 1-heptad linker endpoint reported by Ellis et al. [170]: (i) overview of PDB 8UR5 with EMD-42482 and (ii) a local linker inset. (B) Corresponding renderings of the 6-heptad linker endpoint: (i) overview of PDB 8UR7 with EMD-42486 and (ii) a local linker inset. Panels A and B were rendered with identical camera, crop, contour, and visual-encoding settings. They support realization of different local helical linker endpoints, not quantitative whole-particle center-to-center spacing, peptide presentation, T-cell causality, or vaccine efficacy. The authors generated the molecular views from deposited PDB/EMDB data using UCSF ChimeraX [171]; the repository data are available under CC0 1.0 (https://creativecommons.org/publicdomain/zero/1.0/), and no published Ellis figure artwork was reproduced or adapted. The structures and linker endpoints were reported by Ellis D, Dosey A, Boyoglu-Barnum S et al., “Antigen spacing on protein nanoparticles influences antibody responses to vaccination,” *Cell Reports* 42, 113552 (2023), https://doi.org/10.1016/j.celrep.2023.113552. (C) AlphaFold model assessment from Terwilliger et al. [172] for PDB 7S5L. (i) A high-confidence AlphaFold prediction (magenta) compared with the corresponding experimental electron-density map in a region containing an incorrect local prediction. (ii) The deposited experimental model (blue) compared with the electron-density map in the corresponding region. The comparison shows that experimental density can adjudicate local model fit; it does not validate the Ellis-derived panels A or B and does not test delivery, antigen presentation, immune function, or efficacy. Panel C is adapted from source Fig. 1b and f in Terwilliger TC, Liebschner D, Croll TI et al., “AlphaFold predictions are valuable hypotheses and accelerate but do not replace experimental structure determination,” *Nature Methods* 21, 110–116 (2024), https://doi.org/10.1038/s41592-023-02087-4, under the Creative Commons Attribution 4.0 International License (https://creativecommons.org/licenses/by/4.0/). Panels A–C do not determine carrier- or junction-derived pMHC identity.

## 5 Cross-cutting constraints, design responses, and evidence synthesis

### 5.1 Repeat dosing is a functional, not merely serological, question

Anti-carrier responses can alter PK, uptake, or tolerability. Some PEGylated liposomes show formulation-, dose-, and interval-dependent accelerated blood clearance [[173], [174], [175]], but seropositivity alone is not failure. In one mRNA-vaccine study, induced anti-PEG antibodies did not affect the assessed neutralizing response [79]. A PK study followed 19 adults, each previously given three or four monovalent doses, through one Moderna bivalent booster. Baseline anti-PEG antibodies did not track mRNA or decay of the ionizable lipid SM-102; peak mRNA and SM-102 exposures were associated with subsequent IgG and IgM expansion, respectively [80]. This small single-booster study does not establish repeat-dose behavior in therapeutic cancer vaccination.

In a preventive mouse human papillomavirus (HPV) model, controls had 19-fold higher HPV16- and HPV18-neutralizing titers than mice preimmunized with *Pyrococcus furiosus* ferritin before a ferritin-displayed L2-polytope vaccine [176]. The study neither measured PK nor isolated antibodies from broader anti-scaffold immunity and is not human evidence in therapeutic cancer vaccination.

Viral vectors are boundary comparators because neutralization is platform specific. Four weeks after recombinant adenovirus type 5 (rAd5) boosting in the human immunodeficiency virus (HIV) Vaccine Trials Network (HVTN) 204, the Gag-specific CD4^+^ T-cell responder rate was 12% (7/57) in participants with pre-existing Ad5 fiber-directed neutralizing antibodies versus 39% (25/64; *P* < 0.001) without them; magnitude comparisons did not pass the prespecified multiplicity threshold [177]. Yet pre-existing immunity did not always preclude response. In a 32-patient adenovirus 5-carcinoembryonic antigen (Ad5-CEA) study, 19/31 tested patients had baseline neutralizing activity, but CEA-specific enzyme-linked immunosorbent spot (ELISpot) responses did not differ by the authors' antibody stratum [178]. That uncontrolled study cannot establish efficacy or independence from pre-existing immunity.

In the EVOLVE trial of intravenous enadenotucirev, infectivity was detected in 14/14, 12/15, and 3/12 post-infusion samples at cycles 1, 2, and 3, while anti-enadenotucirev titers rose. Changing denominators and the observational design prevent attributing the decline to antibodies or inferring impaired tumor delivery [179]. T-VEC is administered intralesionally; its label reports 58% baseline herpes simplex virus type 1 (HSV-1) seropositivity without a serostatus-stratified efficacy estimate [106]. The non-replicating VLP and protein-cage vaccine systems in this evidence set do not mediate vector transduction; recognition may instead alter masking, uptake, or competition [[91], [92], [93], [94]]. The relevant endpoint is later-dose delivery, antigen-specific functional response, or tolerability, not seropositivity alone.

### 5.2 Carrier-directed immunity can help, coexist, or interfere

Carrier visibility matters only when recognition changes exposure or antigen-specific function. Intrinsic innate activity remains distinct: empty LNPs can activate without encoded antigen [32], and membrane carriers retain source-dependent cues [39,40,[68], [69], [70]]. Cross-platform magnitudes are not comparable across different doses, routes, and assays; attribution requires empty-carrier, antigen-only, adjuvant-only, and matched controls.

Carrier-directed helper responses can be substantial. After two eOD-GT8 60-mer doses in the prophylactic International AIDS Vaccine Initiative (IAVI) G001 trial, eOD-GT8-specific and lumazine-synthase-specific CD4^+^ T-cell responses were detected in 84% and 93% of recipients; 85% recognized a scaffold hotspot [90]. In two mouse strains after one ferritin-hemagglutinin immunization, ferritin peptides accounted for 85% and 88% of summed interleukin-2 and 98% and 99% of summed IFN-γ ELISpot responses. Separate experiments found that ferritin-specific cells comprised 85% and 87% of responding T follicular helper cells and that hemagglutinin-specific germinal center B cells were 3.7- and 3.8-fold more abundant than after equimolar hemagglutinin trimer [95].

These data allow coexistence or intramolecular help but do not prove benefit in therapeutic cancer vaccination. The studies concern prophylactic HIV vaccination and murine influenza, and the ferritin comparison also changes valency and architecture; the B-cell increase cannot be assigned to scaffold-specific help alone. Carrier-directed immunity is helpful, neutral, or harmful only through measured effects on first-dose priming, later-dose delivery, target-antigen function, or tolerability.

Because fusion junctions recur when tumor antigens change, pMHC complexes containing junction-derived peptides are a testable risk rather than an established outcome. Prediction can prioritize recurrent junctions [[180], [181], [182]]. Available assays answer different questions: a major histocompatibility complex class II (MHC-II) MAPPs assay detects presented peptides [183], whereas standalone B-cell and dendritic-cell activation assays provide ancillary risk information [184,185]. Therapeutic-protein literature distinguishes tolerance or mitigation strategies [186] from nonclinical and clinical immunogenicity risk frameworks [187,188]; both can inform triage but do not establish junction presentation.

Direct presentation can be assessed with MHC class I (MHC-I) immunopeptidomics or additional MHC-II MAPPs methods [133,143,144,189,190], and junction-specific T-cell assays then test functional recognition [191]. Standalone activation assays cannot by themselves demonstrate junction-derived pMHC or competition [184,185,192]. No single assay establishes clinical immunogenicity; a junction-risk claim requires direct peptide detection together with a demonstrable change in target-antigen-specific function. The goal is preserved antigen-specific function, not zero carrier immunogenicity (Fig. 7B).

### 5.3 Personalized manufacture, lot release, consistency, and post-change comparability

Manufacturing can confound biological attribution. Potential product-specific critical quality attributes (CQAs) include size, antigen copy number and orientation, loading, release, ligand density, impurities, and potency. For sequence-specific mRNA manufacture, development may need payload identity, integrity, stability, and potency [81]; LNP development may additionally require particle-size distribution or polydispersity, encapsulation or loading, lipid composition, stability, and potency [82]; protein cages may require assembly, antigen copy number and orientation, aggregation, stability, and potency [96,97]; and EVs may require identity, purity, source-cell attributes, storage stability, and potency [87,88]. These are product-development and characterization examples, not a claim that every item is a routine release test. Product-specific methods are needed to assess EV identity [87,88], conjugation site [157], peptide presentation [144,189,190], cellular responses [191,192], and host-cell protein impurities [193]. No universal threshold spans LNPs, cages, polymers, and EVs; acceptance criteria require development data, data from clinical and consistency lots, and stability data.

Personalization creates repeated sequence-specific products. A small-scale good manufacturing practice (GMP) process produced reproducible neoantigen mRNA for a dendritic-cell-vaccine program but did not test LNPs or V940 [81]. A 128-channel microfluidic system increased LNP throughput while preserving tested properties and preclinical activity [82], but cannot establish another product's cost, consistency, or comparability.

Routine lot release, lot-to-lot consistency, and post-change comparability are distinct. Where International Council for Harmonisation (ICH) Q5E applies to qualifying products from one manufacturer, pre- and post-change products need not be identical, but differences must not adversely affect quality, safety, or efficacy [194]. Q8–Q11 support development and lifecycle control, whereas Q5C and Q6B concern characterized protein or polypeptide products; Q6B excludes conventional vaccines, DNA products, and cells. Applicability to mRNA-LNPs, EVs, or individualized vaccines depends on product classification and regulatory consultation.

A billion-dose-per-year COVID-19 model estimated production plus fill-finish costs of US$0.54 per adenoviral vector dose in 10-dose vials and US$2.39 and US$0.39 per conventional mRNA and self-amplifying RNA dose in 5-dose vials [195]. These are model outputs, not observed costs, and cannot be transferred to individualized cancer-vaccine courses or affordability.

Cross-platform costs lack a common product, scale, release package, failure assumption, and treatment-course denominator. LNPs require lipid formulation and cold-chain control; cages require expression, assembly, and aggregation testing; EVs add producer-cell and separation constraints; personalized products require repeated small-lot release testing. A defensible ranking must normalize these platform-specific burdens together with the functional consequences and decision gates summarized in Table 4.

**Table 4:** Platform constraints, functional consequences, evidence requirements, and decision gates.

Constraint	Potential functional consequence	Evidence needed	Design response or decision gate
Anti-carrier antibodies or complement activation [30,80,175]	No effect, altered clearance or uptake, or added reactogenicity	Anti-carrier antibody titers, complement-activation markers, later-dose PK and biodistribution	Retain only if later-dose delivery and antigen-specific function remain within justified limits
Carrier-specific CD4^+^ T cells [90,95]	Cognate help, coexistence, or epitope competition	Paired carrier- and antigen-specific CD4^+^ responses, including Tfh; antigen-specific B- or T-cell function	Preserve helpful responses; redesign only when carrier-specific immunodominance impairs the target response
Potential scaffold-, linker-, or junction-derived peptides [133,181,183,191]	Possible help or competition, not established without direct presentation and functional assays	Prediction followed by MHC-I immunopeptidomics or MHC-II MAPPs and junction-specific functional T-cell assays	Consider sequence redesign when presentation and disruptive function are demonstrated
Within-lot heterogeneity or lot-to-lot variability [81,82,88,194]	Variable loading, release, tissue access, or potency	Product-specific CQAs, a qualified potency assay, within-lot distribution data, and lot-to-lot consistency data	Use development- and stability-justified limits; reject out-of-specification lots
Personalized antigen update or manufacturing change [81,194]	Rapid adaptation, repeated small-lot burden, and possible CQA shifts	Sequence identity, turnaround, dose, potency, same-product lot-to-lot consistency when repeated lots exist, process consistency across individualized batches, and post-change comparability when applicable	Use only if sequence-specific lot-release testing, applicable process-consistency evidence, and post-change comparability testing fit the clinical schedule

*Note:* Sources support observed immune effects, assay capabilities, or quality principles; decision gates are this review's synthesis. ICH guidance applies according to product scope and regulatory context; Q6B does not cover conventional vaccines, DNA products, or cells [194].

*Abbreviations:* CQA, critical quality attribute; MAPPs, major histocompatibility complex-associated peptide proteomics; MHC, major histocompatibility complex; PK, pharmacokinetics; Tfh, T follicular helper cell.

**Table 5:** Evidence status of selected variable-to-outcome comparisons.

Variable and matched comparison	Delivery or product link	Antigen-specific outcome and boundary	Status; next measurement
LNP formulations: optimized 113-O12B versus ALC-0315 [41]	Draining-node and liver expression differed; APC transfection compared ALC-0315 with two 113-O12B PEG ratios	SIINFEKL-responsive IFN-γ^+^ and H-2K^b^–SIINFEKL tetramer-positive CD8^+^ T cells, with prophylactic and therapeutic B16F10-OVA outcomes	Partial; the optimized-formulation comparison did not independently isolate lipid identity or delivery-to-tumor mediation
Phenylboronic ester–modified versus unmodified PEG-b-PAsp/TRP2 formulation [34]	Modification altered stimulus-responsive release, uptake and localization in dendritic cells, and draining-node retention	Total CD8^+^ T-cell readouts, frequencies of CD44/CD62L-defined memory T-cell subsets after ex vivo TRP2 restimulation, and B16F10 outcomes; no direct TRP2-derived pMHC or TRP2-specific T-cell frequency	Partial; quantify TRP2-derived pMHC and TRP2-specific T cells under the matched comparison
Particulate long peptide (LSP) versus soluble counterpart (LSS), matched adjuvant [46]	LSP-7/8a showed greater draining-node retention and uptake by CD11c^+^ cells than LSS-7/8a	Higher OVA-specific CD8^+^ T-cell responses and improved B16.OVA challenge outcome	Partial; the LSP/LSS and chimeric controls support a physical-form contribution, but flanking sequences differed and processing-related effects were not independently separated; further isolate physical form from sequence and conjugation effects
hFTN-RFP versus free RFP, empty hFTN, and phosphate-buffered saline [152]	Greater lymph-node targeting and retention; APC uptake unmeasured	RFP-peptide-restimulated IFN-γ^+^CD8^+^ T cells per 10^5^ splenocytes and prophylactic B16F10-RFP control	Partial; test antigen-copy equivalence, APC uptake or pMHC, and established-tumor efficacy
Defined eOD-GT5 constructs with valencies of 1, 4, 8, and 60 [142]	Valency-associated changes in cognate B-cell activation, proliferation, differentiation into germinal center B cells and plasma cells, and response composition	Cognate B-cell outcomes in prophylactic HIV-immunogen models; no therapeutic cancer-vaccine test	Partial; prophylactic context; architecture, epitope accessibility, and density were not fully matched
Endosomolytic polymersome-encapsulated versus free cGAMP and carrier controls [86]	Encapsulation increased fluorescent cyclic-dinucleotide-tracer uptake and cGAMP-driven STING activity; enhanced cytosolic delivery was inferred	No selected antigen co-loaded or antigen-derived pMHC measured; tumor control and rechallenge protection were observed	Untested for antigen co-delivery; measure intracellular routing, antigen-derived pMHC, and antigen-specific function
Genetic fusion and site-specific conjugation [[157], [158], [159], [160]]	Antigen attachment and nanoparticle characterization [[158], [159], [160]]; site-specific bioconjugation strategies are reviewed in [157]	Antigen-specific IFN-γ^+^CD8^+^ T-cell responses and tumor control in mice [158]; vaccine responses [159,160], including anti-ferritin antibodies [160]. These outcomes do not establish junction-derived pMHC presentation or its effects on target-antigen-specific T-cell function	Untested for junction-specific T-cell immunodominance; establish junction-derived pMHC presentation and its effects on target-antigen-specific function

Table 5 classifies selected comparisons according to whether a matched variable-to-delivery-to-antigen-specific chain is complete in the tested model. The cases illustrate specific claims and do not constitute a systematic assessment of the field. Each status applies to the stated comparison, model, outcome, and available evidence. **Supported** requires the complete chain; **Partial** denotes an incomplete or context-limited chain; **Untested**, a missing decisive experiment; and **Refuted**, a stated generalization contradicted within the specified comparison scope. Surrogate readouts and multicomponent comparisons are not treated as single-variable demonstrations.

*Abbreviations:* APC, antigen-presenting cell; cGAMP, 2′3′-cyclic GMP–AMP; hFTN, human ferritin heavy chain; HIV, human immunodeficiency virus; IFN-γ, interferon-γ; LNP, lipid nanoparticle; LSP, particulate long peptide; LSS, soluble long peptide; 7/8a, Toll-like receptor 7/8 agonist; OVA, ovalbumin; PAsp, poly(aspartic acid); PEG, polyethylene glycol; pMHC, peptide–major histocompatibility complex; RFP, red fluorescent protein; STING, stimulator of interferon genes; TRP2, tyrosinase-related protein 2.

### 5.4 Evidence synthesis across selected variable-to-outcome chains

The 113-O12B comparison links formulation changes to altered lymph-node and liver expression, alongside antigen-responsive T-cell and tumor readouts [31]. The formulation-level comparison does not independently isolate the contribution of lipid identity or establish how the distribution change mediated the tumor outcome. Matched-adjuvant and chimeric-peptide controls in the LSP/LSS study support a contribution of particulate presentation to lymph-node retention and antigen-specific immunity [46]. Differences in flanking sequence leave processing-related effects unresolved and limit attribution of the full benefit to physical form alone. Both comparisons retain positive evidence of delivery-related benefit; their Partial classifications identify different limits to attribution. A formulation-level gain can justify further evaluation of that formulation. Generalization to other formulations using the same lipid or to other particulate peptide designs requires additional evidence that isolates the contribution of the relevant carrier feature. These distinctions help identify the next unresolved comparison without treating an incomplete causal chain as absence of biological activity.

## 6 Artificial intelligence-assisted design: bounded triage with explicit experimental gates

Repeat-dose performance, carrier-directed immunity, manufacturing, and scheduling constrain platform design. Artificial intelligence (AI) can narrow the candidate space for carriers, sequences, fusions, or formulations (Fig. 9A and B), but outputs remain hypotheses subject to domain, product, delivery, and function/toxicity gates; the product gate includes a product-relevant potency endpoint. Prediction scores do not establish delivery, antigen-specific immune function, or clinical benefit.

**Fig. 9:**
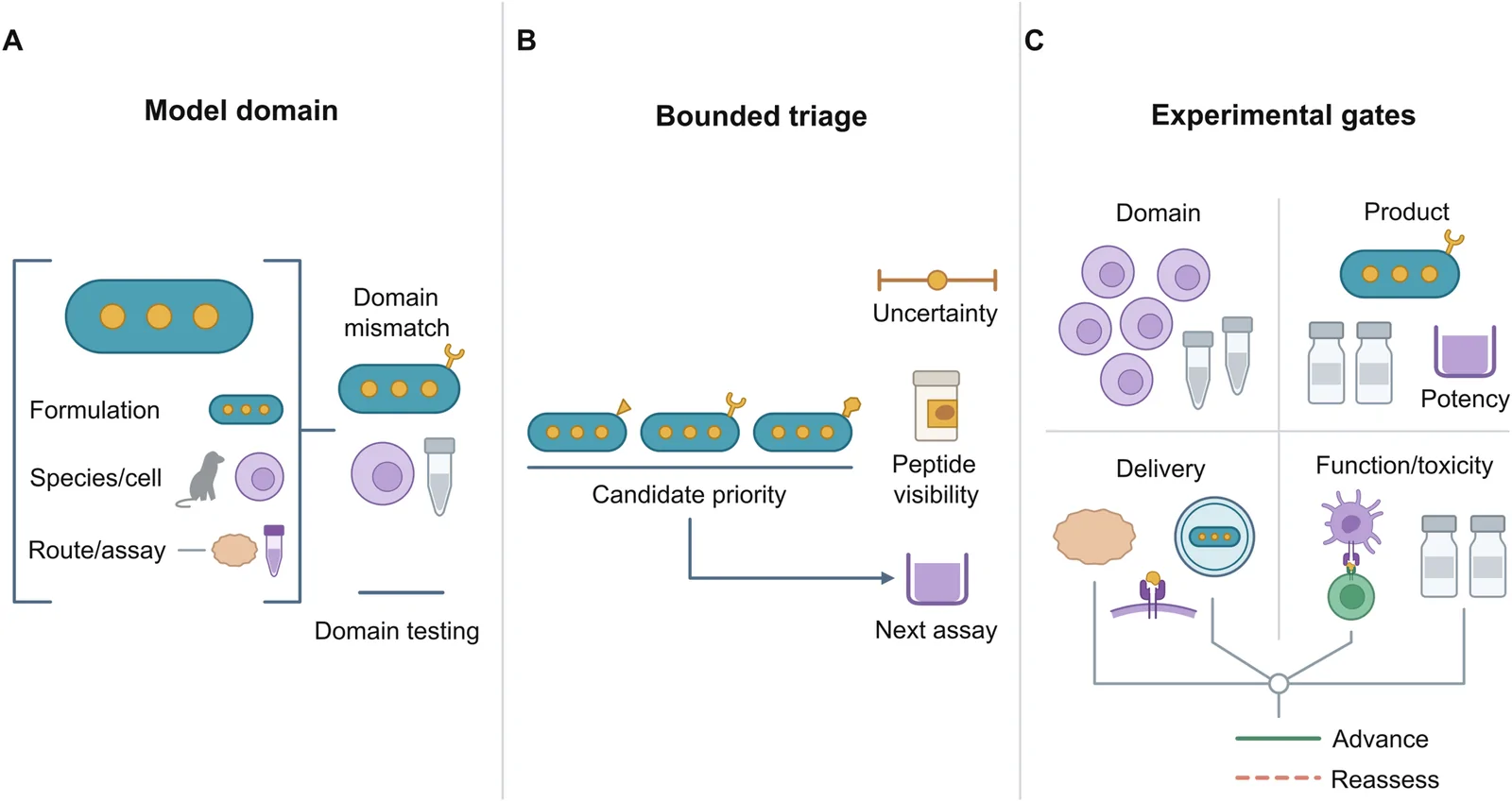
Bounded model-domain triage must terminate in experimental gates. (A) The model domain is defined by the formulation, species or cell system, and route or assay; a domain mismatch triggers testing rather than automatic rejection. (B) Bounded triage supports candidate prioritization, uncertainty communication, peptide-visibility assessment, and selection of the next assay; prediction is not evidence of delivery or efficacy. (C) Experimental gates evaluate domain, product attributes including potency, delivery, and function/toxicity before conditional advance or reassessment. Created in BioRender. Wang, S. (2026) https://BioRender.com/1meeco7.

### 6.1 Decisions that computation can prioritize

AI triage should begin with a defined delivery bottleneck and antigen format. Peptide–HLA models [181], immunopeptidomics [196,197], and clonality or neoantigen-fitness analyses [198,199] can prioritize payloads; mutant MHC-II response data provide a functional anchor [200]. Structure-prediction and protein-design models can prioritize structure or sequence candidates [[201], [202], [203], [204], [205]], Bayesian optimization prioritizes formulation [206], and computational dose optimization prioritizes regimen candidates [207]. Trial-design methods can organize downstream validation [208]. None of these outputs constitutes carrier evidence or resolution of the target bottleneck without prospective matched experiments.

For protein scaffolds, models can prioritize fusion sites, linker lengths, and candidate junction peptides [181,[209], [210], [211]], while uncertainty-aware immunogenicity and deimmunization frameworks inform risk triage [[212], [213], [214], [215], [216]]. A claim of junction-peptide-mediated interference requires direct evidence that candidate-derived peptides are presented by MHC/HLA molecules and change target-antigen-specific function. Surface accessibility and assembly require separate characterization. For lipid, polymer, or EV systems, computation can prioritize compositions or source-cell features, but PK, innate sensing, tissue distribution, and source-cell effects remain experimental [39,40,68,69].

### 6.2 Domain shift and proxy failure

Model failure can arise from the choice of training labels. Delivery rankings for 281 LNPs in cultured endothelial cells and macrophages did not predict their rankings across tissues *in vivo* [217]. A separate 89-LNP comparison found species-dependent functional mRNA delivery and cellular responses involving translation and endocytosis [218]. A model trained on one cell line, species, route, or transfection endpoint may therefore be internally accurate but lack relevance to the intended therapeutic endpoint.

Structure models present a different proxy limitation. In adversarial tests of 200 proteins, AlphaFold 3 retained the same global fold on average after disruptive mutations affecting up to 40% of residues, and its confidence scores selected the most accurate structure in no more than 35% of comparisons; ESMFold was more mutation-sensitive but remained imperfect [219]. Although not a cancer-vaccine delivery study, this result shows why structural confidence cannot replace assembly, surface accessibility, or processing data. Experimental density in Fig. 8C adjudicates the structural model; the underlying study does not assess delivery. Transformer-guided LNP design likewise produced experimentally validated candidates without uniform *in vivo* improvement [220], so prospective experiments determine utility.

### 6.3 Claim-matched validation and experimental gates

These failures motivate four experimental decision gates for advancing or reassessing an AI-prioritized design, each addressing a distinct failure mode. The domain gate is unmet when the candidate lies outside the chemistry, sequence, species, cell type or cell line, route, endpoint, or assay context represented in the training data and no relevant external validation in the target domain is available. Independently, the product gate is unmet when the resulting product or lot fails applicable, prespecified criteria for identity, assembly, loading, stability, potency, or surface accessibility, or when the manufacturing process fails applicable lot-to-lot consistency or post-change comparability criteria. The delivery gate is unmet when the predicted advantage does not improve the prespecified trafficking, release, or pMHC endpoint under matched controls, whereas the function/toxicity gate is unmet when the candidate does not improve antigen-specific immune function at an acceptable safety margin. An unmet gate triggers reassessment, redesign, deprioritization, or discontinuation rather than automatic advancement.

Single-cell and multimodal profiling can explain candidate performance [221,222], but exploratory profiles should not replace a prespecified potency endpoint. Validation must match the claim: structural characterization for geometry, *in vivo* distribution for targeting, direct measurement of presented peptides for processing, and antigen-specific function for a therapeutic-vaccine claim (Fig. 9C). Table 6 applies these gates to common design decisions.

**Table 6:** AI-assisted triage and experimental gates.

Decision	AI triage output	What AI cannot establish	Required evidence	Experimental-gate category
Training-domain applicability [217,218]	Training-domain metadata plus an out-of-distribution (OOD) flag for chemistry, sequence, species, cell type or line, route, endpoint, and assay	Transferability to an unrepresented domain	External validation in the target domain before advancement; otherwise defer advancement pending validation	Domain
Upstream payload prioritization [181,197,199]	Antigen ranking by predicted presentation or clonality for payload selection	Tumor clonality, natural processing, presentation, or T-cell recognition	Tumor sampling and variant evidence for clonality, plus immunopeptidomics and an antigen-specific T-cell assay for processing, presentation, and recognition	Delivery and function/toxicity
Carrier or formulation choice [218,220]	Ranked compositions for a prespecified endpoint	Cross-species pharmacokinetics, biodistribution, innate sensing, or efficacy	Relevant *in vivo* delivery, product-relevant potency, and safety comparison	Domain, product, delivery, and function/toxicity
Fusion or scaffold design [[201], [202], [203],^219^]	Predicted assembly, surface accessibility, or steric clash	Product assembly, proteolytic processing, formation and cell-surface display of candidate-derived pMHC complexes	Biophysical characterization plus product-relevant potency and a claim-matched antigen-display or pMHC assay	Product and delivery
Platform-derived peptide risk [133,181,183,191]	Ranked scaffold, linker, or junction peptides	Immunodominance or harmful competition	MHC-I immunopeptidomics or MHC-II MAPPs, plus paired carrier- and antigen-specific responses in one assay, with absolute values and detection limits; ratios secondary	Delivery and function/toxicity
Payload ratio or release profile	Reduced experimental search space (formulation-search precedent [206])	Co-distribution, temporal coupling, or therapeutic benefit	Co-loading, release kinetics, product-relevant potency, APC activation state, and antigen-specific function [34,46]	Product, delivery, and function/toxicity

*Note:* Sources support prediction tools, failure examples, or experimental methods; the four-gate scheme is this review's synthesis. Reference [206] optimizes formulation stability, not antigen–adjuvant ratios or release kinetics, which require direct measurements.

*Abbreviations:* AI, artificial intelligence; APC, antigen-presenting cell; MAPPs, major histocompatibility complex-associated peptide proteomics; MHC, major histocompatibility complex; OOD, out of distribution; pMHC, peptide–major histocompatibility complex.

## 7 Conclusions and outlook: evidence-gated delivery design

No carrier is optimal across all bottlenecks. Platform selection requires a defensible match among the dominant bottleneck, the variable changed, and the evidence used to advance a product. A carrier advantage is supported only when a defined variable changes a prespecified transport, release, or processing step, or a verified spatially or temporally coupled immune-modulation step, and that change is linked to an antigen-specific outcome. Increased uptake or tumor regression alone is insufficient.

Evidence strength must match the claim. Geometric principles from preventive vaccines, drug-encapsulation studies, and *in situ* tumor vaccination can inform mechanism, but none proves delivery of a preselected therapeutic cancer antigen. Products must remain functional across doses and independently manufactured lots. Carrier-directed immunity matters only if it changes later-dose PK or antigen-specific immunity; human LNP evidence informs anti-PEG recognition and post-booster exposure [79,80], while protein-scaffold studies address scaffold-specific CD4^+^ responses and antibodies [90,92,95] as well as pre-existing-carrier effects or epitopic suppression [91,93,94]. Routine lot release, lot-to-lot consistency, applicable post-change comparability, and clinical scheduling should be evaluated alongside delivery performance because they determine translational feasibility, especially for patient-specific antigen sequences.

Future studies should use prespecified matched comparisons that control antigen dose, adjuvant, route, and product quality; factorial or dose-matrix designs are preferable when interactions are plausible. Such experiments should link biophysical measurements to tissue distribution, surface pMHC abundance, and antigen-specific function at initial and later doses. Cross-study comparability would improve if immunopeptidomics [133,143,144], paired carrier- and antigen-specific responses measured within the same assay, and lot-level CQAs were reported with absolute values and detection limits, using ratios only as secondary summaries. AI may prioritize these experiments, but advancement remains governed by the experimental gates in Section 6.3.

A material modification has not produced a translational delivery advantage if it fails to improve its prespecified delivery endpoint, loses the effect after boosting, or cannot be reproduced across independently manufactured lots meeting predefined acceptance criteria. The same criterion can be applied to synthetic, biological, and protein-based systems while preserving their distinct evidentiary and regulatory boundaries.

## CRediT authorship contribution statement

**Shuai Wang:** Writing – review & editing, Writing – original draft, Visualization, Supervision, Investigation, Conceptualization. **Mai Zhang:** Writing – original draft, Visualization, Investigation. **Jing Tian:** Writing – review & editing, Investigation. **Jiong Wu:** Writing – review & editing, Investigation. **Shentao Lu:** Writing – review & editing, Supervision, Conceptualization. **Lubin Liu:** Writing – review & editing, Supervision, Conceptualization. **Dingkang Liu:** Writing – review & editing, Supervision, Funding acquisition, Conceptualization.

## Ethics approval and consent to participate

This review is based on previously published literature and data. No new studies involving human participants, human data, human tissue, or animals were performed by the authors for this work. Therefore, ethics approval and consent to participate were not required.

## Declaration of generative AI and AI-assisted technologies in the manuscript preparation process

During preparation of this work, the authors used a large language model for language editing and reference-metadata checking. The tool was not used to create primary data or quantitative results. The authors independently checked the cited sources, reviewed and edited the generated content, and take full responsibility for the content of the published article.

## Funding statement

This work was supported by the National Natural Science Foundation of China [grant numbers 82674905 and 82304368] and the Chongqing Medical Scientific Research Project (Joint Project of Chongqing Health Commission and Science and Technology Bureau) [grant number 2023DBXM001].

## Declaration of competing interest

The authors declare that they have no known competing financial interests or personal relationships that could have appeared to influence the work reported in this paper.

## Data Availability

No data was used for the research described in the article.
