# Supplementary material for: A bottleneck-first evaluation of antigen delivery platforms for therapeutic cancer vaccines
Source: Mater Today Bio. 2026 Sep 30;41:103715. doi: 10.1016/j.mtbio.2026.103715 (PMC13659479; doi:10.1016/j.mtbio.2026.103715)
Supplement: Supplementary file 1 [file mmc1.docx]

**Supplementary Information**

Reference numbers correspond to those in the main manuscript.

# **Figure S1. Preclinical and clinical examples of vaccination combined with checkpoint modulation at the regimen level.**

(A–C) Preclinical regimen-level evidence from Kim et al. [222]. (A) B16F10 study and sampling design for five treatment arms—control, poly(I:C), poly(I:C) plus a tier-1 synthetic-long-peptide vaccine, poly(I:C) plus anti-PD-1, and the triple combination—with spleen and tumor sampling for single-cell RNA and T-cell-receptor sequencing. (B) Tumor volume over time in the five treatment arms. (C) Frequencies of IFN-γ^+^CD8^+^ splenic T cells after restimulation with the displayed tier-1 neoepitopes. The vaccine consisted of synthetic long peptides with poly(I:C), not a nanocarrier. The treatment arms permit conditional assessment of vaccine and anti-PD-1 contributions on a common poly(I:C) background; they do not establish a carrier-specific delivery mechanism or physical co-delivery. Panels A–C are adapted from source Supplementary Figs. 7a, 7c, and 7d in Kim SH, Lee BR, Kim SM, et al., “The identification of effective tumor-suppressing neoantigens using a tumor-reactive TIL TCR-pMHC ternary complex,” *Experimental & Molecular Medicine* 56, 1461–1471 (2024), https://doi.org/10.1038/s12276-024-01259-2, under the Creative Commons Attribution 4.0 International License (https://creativecommons.org/licenses/by/4.0/).

(D–E) Clinical regimen-level evidence from Yarchoan et al. [1]. (D) Manufacturing workflow and clinical dosing design for the individualized DNA vaccine GNOS-PV02 plus pIL12 and pembrolizumab. (E) Paired pretreatment and each participant’s highest posttreatment cumulative IFN-γ enzyme-linked immunosorbent spot response to positive vaccine-encoded neoepitopes in 22 participants, reported as spot-forming units per 10^6^ cells. This single-arm phase 1/2 study evaluated a multicomponent regimen; the paired immune-response change and clinical outcomes cannot be attributed to the vaccine alone, and the panel is not a matched efficacy comparison. Panels D–E are adapted from source Figs. 2a and 3a in Yarchoan M, Gane EJ, Marron TU, et al., “Personalized neoantigen vaccine and pembrolizumab in advanced hepatocellular carcinoma: a phase 1/2 trial,” *Nature Medicine* 30, 1044–1053 (2024), https://doi.org/10.1038/s41591-024-02894-y, under the Creative Commons Attribution 4.0 International License (https://creativecommons.org/licenses/by/4.0/). Panels A–C and D–E derive from independent studies of systemic co-administration; neither demonstrates physical co-delivery.
